# Serum Trace Elements and Concurrent Renal, Hepatic, and Electrolyte Biomarkers: A Secondary Cross-Sectional Analysis of SPES

**DOI:** 10.3390/ijms27209313

**Published:** 2026-10-20

**Authors:** Pellegrino Cerino, Fabrizio Minichilli, Elvira De Rosa, Maria Triassi, Paolo Montuori, Carlo Buonerba

**Affiliations:** 1 Department of Public Health, University of Naples “Federico II”, Via Sergio Pansini 5, 80131 Naples, Italy; pellegrinocerino@gmail.com (P.C.); derosaelvira92@gmail.com (E.D.R.); triassi@unina.it (M.T.); paolo.montuori@unina.it (P.M.); 2 Institute of Clinical Physiology (IFC), National Research Council (CNR), Via G. Moruzzi 1, 56124 Pisa, Italy; fabrizio.minichilli@cnr.it; 3 Department of Human Sciences and Quality of Life Promotion, San Raffaele University, 00166 Rome, Italy

**Keywords:** human biomonitoring, serum trace elements, SPES, creatinine, liver enzymes, uric acid, ferritin, electrolytes, cross-sectional study

## Abstract

The relationships between circulating trace elements and routinely measured renal, hepatic, and electrolyte biomarkers remain incompletely characterized. We conducted a secondary cross-sectional analysis of 19 serum elements and concurrent biochemistry in 4137 adults from the Exposure Study on Susceptible People (SPES), Campania, Italy. Creatinine, alanine aminotransferase (ALT), gamma-glutamyl transferase, and potassium were primary outcomes; additional renal, hepatic, mineral, and protein-related measurements were secondary or exploratory. Associations were estimated with covariate adjustment, multiple imputation, and within-family false-discovery-rate control. Per doubling of concentration, lithium was associated with 0.54% higher creatinine (95% confidence interval, 0.22–0.86%). Antimony associations with ALT and potassium were sensitive to the handling of below-quantification concentrations and were not established among directly quantified measurements. Secondary and exploratory findings included higher bilirubin with iron and lower bilirubin with copper; higher ferritin with iron and lower ferritin with cobalt; higher uric acid with strontium, selenium, lithium, and arsenic; and higher albumin, total protein, and total calcium with selenium. Additional albumin adjustment approximately halved the selenium coefficients for total calcium and total protein. The primary associations were small, and their biological and clinical relevance remains uncertain. These cross-sectional findings identify hypotheses concerning renal handling, iron status, and overlapping protein-associated biochemistry.

## 1. Introduction

Multiple factors can influence circulating trace-element concentrations, including external exposure, nutritional status, renal elimination, and distribution among circulating proteins [1,2,3,4]. Essential elements such as iron, copper, zinc, and selenium participate in enzymatic, transport, and redox processes, while excessive exposure to essential or nonessential elements can disrupt biological function [1,2]. Their associations with biochemical measurements can therefore have different explanations: an element may influence physiological function, physiology may influence its circulating concentration, or shared determinants may affect both. Studying these relationships in community populations can inform the interpretation of circulating element concentrations in relation to external exposure and physiological variation.

Routine biochemical measurements provide complementary information about these relationships. Creatinine is widely used to assess kidney function, although muscle mass and diet also influence its concentration [5]. Alanine aminotransferase (ALT) and gamma-glutamyl transferase (GGT) reflect different aspects of hepatic and metabolic biochemistry. In prospective Framingham studies, variation in these enzymes was associated with subsequent metabolic outcomes, including associations within study-defined reference ranges for aminotransferases [6,7]. Serum potassium has also been investigated in community cohorts: higher concentrations were associated with incident stroke in the Malmö Preventive Project, whereas lower concentrations were associated with incident diabetes in the Atherosclerosis Risk in Communities study [8,9]. These observations support studying variation in the circulating measurements themselves, without assuming that an element-related difference in any one marker predicts clinical disease.

Previous human studies have reported selected element–biochemistry relationships, including plasma lithium with estimated filtration, blood lead and urinary cadmium with GGT, and serum iron and copper with bilirubin [3,10,11]. Iron-status indices have also been associated with cobalt in blood and serum [12]. Much of this evidence concerns selected elements or biochemical outcomes in populations with different age and health profiles. A broader panel assessed at the same visit permits related findings and non-findings to be compared within one population. Such comparisons can identify associations consistent with recognized physiology and others for which more specific longitudinal or experimental questions are warranted.

The Exposure Study on Susceptible People (SPES) is a population-based biomonitoring program conducted in Campania, southern Italy. It was developed in a regional setting of heterogeneous environmental pressures and longstanding concern about waste disposal and uncontrolled burning, including the area termed the “Land of Fires” [13,14]. Previous analyses documented geographical variation in circulating elements and associations of seafood consumption with total serum arsenic and mercury [15,16]. A related blood-biomarker analysis reported selected associations with lipid and blood-count measurements, including positive associations of selenium, copper, zinc, and mercury with low-density lipoprotein cholesterol [17]. The present contribution concerns concurrent renal, hepatic, electrolyte/mineral, and protein-associated outcomes rather than these geographical, dietary, or lipid/blood-count questions. It reuses the shared participants, serum assays, and imputation resource; it does not represent a new cohort or new element measurements.

We conducted a secondary cross-sectional analysis to characterize associations between 19 serum elements and concurrent routine biochemistry. Creatinine, ALT, GGT, and potassium formed the primary multiplicity-controlled family, providing a focused set of renal, hepatic, and electrolyte measurements. Additional renal/metabolic, hepatic, and electrolyte/mineral outcomes were secondary; albumin, total protein, globulin fractions, and ferritin formed an exploratory protein transport/storage family. We evaluated the direction and magnitude of associations across the complete element panel, without directional hypotheses for individual pairs, and examined their sensitivity to the handling of low concentrations and model specification. The objective was to identify biochemical relationships and the questions they raise for subsequent research, not to diagnose element-related disease. The primary designation describes the analytical hierarchy of this secondary analysis, not prospectively registered confirmatory hypotheses.

## 2. Results

### 2.1. Participants and Biochemical Measurements

Of 4205 enrolled participants, 17 recruited outside the predefined study area were excluded, leaving 4188 records. Questionnaire and dietary data were available for 4137 participants. Six records were excluded from fully adjusted models because weight, height, or calculated body mass index (BMI) did not satisfy the prespecified data-validity checks, leaving up to 4131 participants (Figure S1). Among participants with questionnaire data, the mean age was 34.9 years (standard deviation [SD], 8.8), and 2236 (54.0%) were women. Median creatinine was 0.80 mg/dL, ALT 35 U/L, GGT 19 U/L, and potassium 4.6 mmol/L. Table 1 presents demographic and biochemical summaries with their observed-data denominators. ALT had an interquartile range of 30–40 U/L and a 95th percentile of 55 U/L. The recorded increments were 0.1 mg/dL for creatinine, 1 U/L for ALT, and 0.1 mmol/L for potassium; the corresponding outcome-resolution analyses are reported in Table S3c. Mercury and nickel primary models included 3350 and 2276 participants, respectively, owing to assay-availability restrictions.

Quantification and assay availability differed across the serum panel. Among the 4137 participants with questionnaire data, lithium had 4110 quantified measurements, 24 below-quantification results, and three observations flagged by the high-concentration plausibility rule. For antimony, 1506 observations (36.4%) were below the limit of quantification (LOQ). Mercury and nickel had 784 and 1860 unavailable assays, respectively. Table 2 summarizes all 19 elements, their concentration distributions, and below-LOQ proportions. Below-LOQ percentages use available assays, whereas concentration summaries describe unflagged quantified measurements. Supplementary Table S1a,b retain the complete assay-state and scaling details.

### 2.2. Primary Element–Biochemical Associations

**Figure 1:**
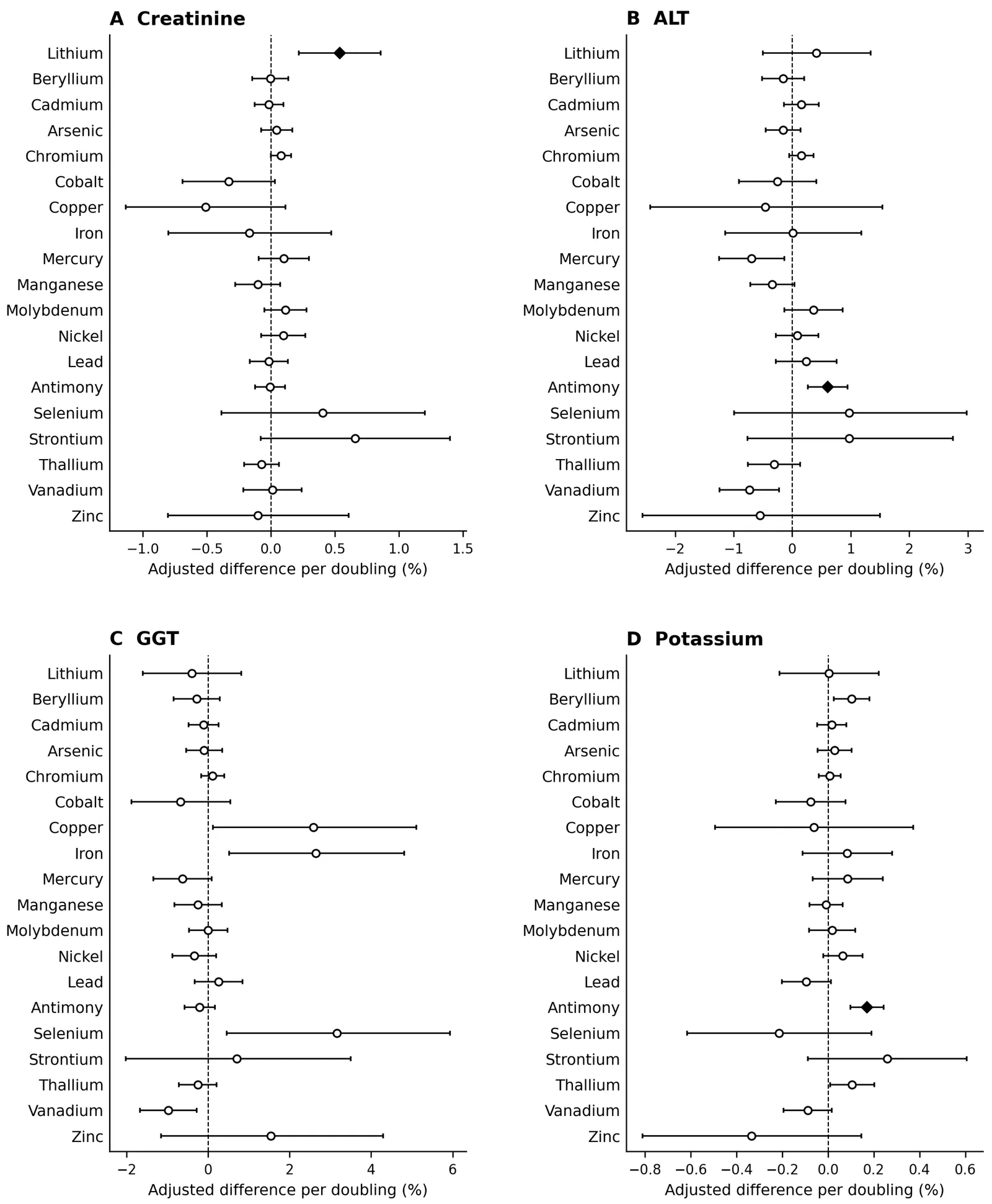
Adjusted primary associations between 19 serum elements and (**A**) creatinine, (**B**) ALT, (**C**) GGT, and (**D**) potassium. Points and horizontal bars denote percentage differences per doubling of concentration and 95% confidence intervals. Filled diamonds identify the three associations meeting q ≤ 0.05 within the complete 76-test family; open circles identify the remaining estimates. Every panel has its own labelled *x*-axis. Mercury and nickel analyses use available-assay subsets. A doubling is a common relative increment, not a common absolute dose or comparative biological potency. Statistical support does not establish biological or clinical relevance; the antimony estimates were sensitive to low-concentration handling (Section 2.4).

Continuous associations are expressed as percentage differences per doubling of serum element concentration. Three of the 76 primary comparisons met the Benjamini–Hochberg false-discovery-rate (FDR) criterion (Figure 1; Table 3). Lithium was associated with 0.54% higher creatinine (95% confidence interval [CI], 0.22–0.86%; q = 0.028), and antimony with 0.60% higher ALT (0.27–0.94%; q = 0.020) and 0.17% higher potassium (0.10–0.24%; q < 0.001). All three models included 4131 participants. No primary linear GGT association met the FDR criterion. Figure 1 presents all 76 estimates, and Supplementary Table S2a–d retain the original fixed log-interquartile-range (log-IQR) results.

At the observed medians, the lithium–creatinine and antimony–potassium percentages correspond to approximately 0.0043 mg/dL and 0.0078 mmol/L, respectively, per doubling. These illustrations are smaller than the recording increments and describe between-person model contrasts, not measured within-person changes or established clinical effects.

For GGT, lead and cadmium were not nominally associated with the outcome (*p* = 0.388 and *p* = 0.557, respectively). Positive nominal associations were observed for iron (+2.64% per doubling; *p* = 0.015; q = 0.146), selenium (+3.16%; *p* = 0.022; q = 0.185), and copper (+2.58%; *p* = 0.040; q = 0.276), and an inverse nominal association for vanadium (−0.98%; *p* = 0.006; q = 0.093). None met the primary FDR criterion. The complete original-scale estimates and confidence intervals are reported in Table S2c.

### 2.3. Secondary Biochemical Associations

Twenty-three of 304 secondary or exploratory comparisons met the FDR criterion within their respective families (Table 4; family definitions in Table S4). All four supported renal/metabolic associations involved uric acid: strontium, 4.24% higher uric acid per doubling (95% CI, 2.70–5.82%); selenium, 1.76% (0.59–2.94%); lithium, 1.35% (0.81–1.90%); and arsenic, 0.50% (0.30–0.70%). None of the urea associations met the 38-comparison renal-family criterion.

Within the hepatic family, iron was positively associated with total and direct bilirubin, whereas copper was inversely associated with both. For total bilirubin, the differences per doubling were +10.01% for iron (95% CI, +4.24% to +16.10%) and −5.31% for copper (−8.26% to −2.27%). Copper was also positively associated with alkaline phosphatase, and vanadium was inversely associated with it. No aspartate aminotransferase (AST) or pseudocholinesterase association met the hepatic-family criterion. In the separate exploratory protein transport/storage family, ferritin was higher with iron (+17.25%; +6.59% to +28.99%) and lower with cobalt (−6.05%; −8.72% to −3.30%).

Selenium was positively associated with albumin (+1.29%; 95% CI, +0.82% to +1.76%) and total protein (+1.37%; +0.88% to +1.86%) in the exploratory protein family and with total calcium (+0.61%; +0.38% to +0.83%) in the electrolyte/mineral family. Phosphate was inversely associated with iron, zinc, and cobalt. Chloride was positively associated with antimony and beryllium and inversely associated with selenium and vanadium; sodium was inversely associated with thallium. All 23 associations are reported in Table 4 under their original testing families. The small selenium–chloride association (q = 0.046) was treated as exploratory and did not support a substantive biological interpretation. Albumin-adjusted selenium sensitivities are reported in Section 2.4. No beta-2 or gamma globulin association met the exploratory-family criterion. Supplementary Results SR1 describe the distribution of findings across the secondary families.

All models include *n* = 4131 and use the Model B adjustment specified in Table 3. Estimates and 95% confidence intervals (CIs) are expressed per doubling of element concentration. q values are Benjamini–Hochberg-adjusted *p* values, calculated separately for renal/metabolic (38 tests), hepatic (95), electrolyte/mineral (76), and exploratory protein transport/storage (95) families. The intervals are not simultaneous multiplicity-adjusted intervals. FDR, false discovery rate; IQR, interquartile range.

### 2.4. Sensitivity Analyses

The lithium–creatinine estimate remained positive across the examined specifications: 0.56% per doubling under alternative censoring completion, 0.69% among directly quantified measurements, and 0.42% with Huber robust regression. Additional adjustment for total protein intake left the estimate at approximately 0.54% per doubling of lithium concentration. Supplementary Figure S2 and Table S11 show the complete profile, including outcome trimming and geographical specifications; Table S8b reports the protein-intake model. Supplementary Results SR2 explain the comparisons. Huber results are point estimates without inferential intervals.

For antimony, the results differed by exposure representation. Under alternative completion, the per-doubling estimates were 0.32% for ALT (95% CI, 0.12–0.52%) and 0.09% for potassium (0.05–0.13%), approximately half the primary estimates. Among directly quantified measurements, they were −0.48% for ALT (−1.39% to +0.44%) and −0.08% for potassium (−0.25% to +0.10%). In contrast, quantified antimony was associated with 5.71% higher ALT (2.71–8.80%) and 1.56% higher potassium (0.93–2.19%) than below-LOQ antimony. These last two estimates compare quantification-status groups rather than a doubling of concentration (Table S8a). The quantified-only analysis included 2622 participants; the quantification-status and alternative-completion analyses included 4126. The comparisons therefore differ in exposure contrast, concentration range, and eligibility. The quantified-only intervals included zero; these results did not establish an inverse effect or confirm a positive continuous gradient.

Outcome trimming retained positive estimates for both primary antimony associations. Under the municipality random-intercept specification, the ALT estimate remained positive but did not meet its sensitivity-family FDR criterion (q = 0.199), whereas the potassium estimate did (q = 0.019; Table S11). Including the concurrent outcome in the alternative completion model yielded similar estimates to completion without it. The secondary antimony–chloride estimate also attenuated under alternative completion; its quantified-only estimate was negative and did not meet the original sensitivity-family FDR criterion (Supplementary Results SR3). Table S12 reports the corresponding comparisons for all 23 secondary or exploratory findings. The potassium assessment involving six erythrocyte-enriched elements identified no positive association meeting the primary FDR criterion (Table S8c). This assessment used existing association results rather than direct specimen-integrity measurements.

In the existing nonrenal sensitivity additionally adjusted for log creatinine, antimony estimates were +0.61% for ALT (95% CI, +0.28% to +0.94%) and +0.17% for potassium (+0.10% to +0.24%), with *n* = 4131. These results did not resolve the censoring-related uncertainty (Supplementary Results SR2).

In additional post hoc analyses, adding natural-log albumin to Model B attenuated the selenium association with total calcium from +0.61% per doubling (95% CI, +0.38% to +0.83%) to +0.29% (+0.08% to +0.50%; nominal *p* = 0.008). The corresponding total-protein estimate decreased from +1.37% (+0.88% to +1.86%) to +0.65% (+0.32% to +0.99%; nominal *p* = 0.00014). The log-scale selenium coefficients were reduced by 53.1% and 52.1%, respectively. All specifications retained the same 4131 participants and 30 completed datasets (Table S13; Supplementary Results SR5).

### 2.5. Complementary Association Models

Global spline tests assess the overall concentration–outcome relationship by considering its linear and nonlinear components together. Seven associations met the separate global-spline FDR criterion: creatinine with lithium and selenium; ALT with manganese and antimony; GGT with iron and selenium; and potassium with antimony. Only the creatinine–selenium nonlinear component met the separately applied nonlinearity criterion (q = 0.028). One of the 76 exposure-by-sex interactions met its FDR criterion: mercury with ALT (q = 0.035), restricted to the available-assay subset. Table S5a summarizes the global and nonlinear tests, and Table S5b reports the interaction contrast; their interpretation is detailed in Supplementary Results SR4.

None of the 32 coefficients from the mutually adjusted eight-element model met the designated FDR criterion (Table S5c). The lithium–creatinine estimate remained positive at approximately 0.49% per doubling (q = 0.088). A simultaneous one-quartile-category increase in lithium, arsenic, cobalt, copper, iron, selenium, strontium, and zinc was associated with 0.79% higher creatinine (95% CI, 0.19–1.39%; q = 0.042). The corresponding ALT, GGT, and potassium mixture estimates did not meet the criterion. The broader 12- and 17-element mixtures were each evaluated within a separate four-outcome family (Table S5d). These joint category contrasts are distinct from the per-doubling element-specific estimates.

## 3. Discussion

Serum trace-element concentrations reflect not only environmental and dietary exposures but also physiological processes involved in their distribution and elimination. Our findings illustrate the importance of these processes when interpreting associations with routinely measured biochemical markers. The lithium–creatinine association was comparatively consistent across sensitivity analyses and was compatible with differences in renal handling, while the iron–ferritin association was consistent with variation in iron status. The selenium associations with total calcium and total protein were substantially attenuated after albumin adjustment, although both remained statistically significant at the nominal level, consistent with overlapping protein-associated variation. By contrast, the antimony associations with ALT and potassium were particularly sensitive to the handling of low concentrations. These findings are discussed below in relation to previous human studies (Table S7).

Renal handling provides a plausible explanation for the lithium–creatinine association, as lower filtration could increase circulating lithium alongside creatinine. Enderle et al. reported an inverse association between plasma lithium and estimated glomerular filtration rate (eGFR) in 928 adults [3]. At the observed creatinine median, our per-doubling estimate corresponds to approximately 0.0043 mg/dL, below the 0.1-mg/dL recording increment. Small between-person contrasts can be estimated across a large sample even when individual values are recorded coarsely; their interpretation remains distinct from a measurable change within an individual. Outcome dispersion and published biological-variation estimates provide further context for this scale difference (Table S3b) [18,19,20,21].

In separate adjusted models, higher serum strontium, selenium, lithium, and arsenic were associated with higher uric acid; all four associations met the renal-family FDR criterion. Xu et al. followed 3957 adults and found that plasma arsenic and strontium were positively associated with incident hyperuricemia and greater annual increases in uric acid [22]. In a cross-sectional analysis of 14,871 NHANES adults, a doubling of blood selenium was associated with 0.119 mg/dL higher uric acid (95% CI, 0.020–0.218) [23]. These comparisons span different matrices, study designs, and outcome scales. Renal clearance remains a plausible shared determinant of circulating lithium, strontium, and antimony. Our uric-acid models did not adjust for creatinine; the filtration restrictions and creatinine-adjusted sensitivities were confined to nonrenal outcomes (Section 4.6).

Iron and copper showed opposite associations with bilirubin, consistent with findings reported by Peng and Wei in 264 healthy adults [11]. In their study, serum iron was positively associated with bilirubin (standardized β = +0.276), whereas serum copper showed an inverse association (β = −0.304; both *p* < 0.001). As their analysis concerned bilirubin values within the reference range, these findings provide a useful context for interpreting physiological variation in bilirubin biochemistry. Although the standardized coefficients cannot be directly compared in magnitude with our percentage differences, the directions of association are consistent. Moreover, because total bilirubin includes the direct fraction, the concordant associations with these two outcomes represent related observations rather than independent evidence.

The positive iron–ferritin association was consistent with variation in iron status, a relationship that may also be influenced by inflammation [24]. Previous studies in menstruating women and adolescents have reported inverse associations between cobalt and ferritin [12,25], highlighting the relevance of iron status when interpreting circulating cobalt concentrations. In the present analysis, inflammation, menstrual blood loss, and supplementation were not directly accounted for in the adjustment set and may have contributed to the observed relationships.

Selenium is distributed among different protein-associated fractions, and total circulating selenium does not necessarily reflect functional selenoprotein concentrations [4,26]. Protein binding is also relevant to the distinction between total and ionized calcium [27]. After additional adjustment for albumin, the selenium coefficients for total calcium and total protein decreased by 53.1% and 52.1%, respectively, although both associations remained statistically significant at the nominal 0.05 level (Table S13). This attenuation is consistent with substantial overlapping albumin-related variation in these measurements. Because albumin is itself a component of total protein, the remaining selenium–total-protein association concerns a composite outcome rather than a separately identified non-albumin fraction. The reduction in coefficients describes attenuation after adjustment without establishing mediation or a direct selenium effect.

No primary linear GGT association met the FDR criterion. In an analysis of 10,098 adults from NHANES III, Lee et al. reported graded positive associations between GGT and blood lead and urinary cadmium concentrations [10]. In SPES, neither serum lead nor cadmium was nominally associated with GGT, although differences in biological matrices, exposure ranges, study populations, and adjustment sets complicate direct comparisons. A serum-based comparison is provided by Peng et al., who reported a positive correlation between copper and GGT in 281 adults attending a physical-examination centre (r = 0.198; *p* = 0.001), which persisted after covariate adjustment [28]. Our copper–GGT association was likewise positive but reached statistical significance only at the nominal level (*p* = 0.040; q = 0.276).

The antimony findings warrant particular attention because their associations with ALT and potassium were sensitive to the handling of low concentrations. With 36.4% of assays below the limit of quantification (LOQ), the positive estimates were approximately halved under alternative completion, while analyses restricted to quantified measurements did not establish corresponding associations. The comparison between quantified and below-LOQ observations represents a contrast between groups rather than a continuous concentration–response gradient. Differences in concentration ranges and participant eligibility, including the exclusion of high-concentration flags, further complicate attribution of these discrepancies to imputation alone. Moreover, the ALT association did not meet its sensitivity-family FDR criterion in the random-intercept model (q = 0.199). Relevant hepatic-metabolic evidence comes from Xie et al., who analyzed 5548 NHANES adults and reported higher odds of metabolic-associated fatty liver disease with increasing dilution-adjusted urinary antimony (OR, 1.18 per natural-log unit; 95% CI, 1.08–1.29) [29]. Their outcome definition combined steatosis assessed by the U.S. Fatty Liver Index with metabolic criteria. This provides a related hepatic-metabolic comparison, although the urinary exposure measurement and composite disease outcome differ from the serum-antimony–ALT association examined here.

Specimen quality is another consideration when interpreting the antimony–potassium association. Potassium measurements can be influenced by collection procedures [30], although neither the available external evidence nor our six-element assessment establishes a preanalytical explanation for the observed findings. Specimen-level hemolysis indices were unavailable, and the element plausibility rule was not designed to assess hemolysis. Such variation could affect measurements of potassium, iron, phosphate, and bilirubin. More reliable quantification of low antimony concentrations, together with direct information on specimen quality, is therefore needed to support the physiological interpretation of the antimony–electrolyte pattern.

The inverse phosphate associations with iron, zinc, and cobalt raise additional questions concerning their physiological interpretation. Previous studies linking iron to fibroblast growth factor 23 (FGF23) and documenting formulation-dependent hypophosphatemia following intravenous iron provide relevant physiological and treatment-related context [31,32], although they do not establish a common pathway underlying the present associations. Similarly, the small selenium–chloride association (q = 0.046) should be considered individually rather than combined with other exploratory electrolyte findings into an untested physiological syndrome.

The common enrollment setting, concurrent biochemical measurements, large sample, and adjustment for individual and dietary characteristics enabled a consistent and precise assessment of associations across the element panel. Nevertheless, statistical precision cannot eliminate systematic measurement bias or residual confounding. The sensitivity analyses provide additional insight into the dependence of individual findings on analytical assumptions, allowing changes in direction and magnitude to be considered alongside differences in statistical significance.

The cross-sectional design remains an important limitation. Because element concentrations and biochemical markers were measured simultaneously, their temporal relationships and the potential contribution of shared determinants cannot be resolved. A single specimen also cannot characterize usual within-person variation. The adjustment set did not directly include C-reactive protein (CRP) or other inflammatory measures, menstrual status or blood loss, or supplement type and dose. Adjustment for sex and selected dietary covariates, together with medication-related restrictions, cannot fully account for these factors. Residual confounding related to body composition, diet, and treatment also remains possible. Furthermore, community recruitment did not establish that all participants were free of disease. The relatively young Campania population and restriction to participants with questionnaire data limit the generalizability of the findings to older adults, patients with established renal or hepatic disease, and populations with different exposure distributions.

Additional limitations concern the measurement and analytical framework. The absence of arsenic and mercury speciation restricts species-specific source attribution and toxicological interpretation, while serum may differ in its suitability as a biomonitoring matrix across elements. Assay unavailability restricted the mercury and nickel analyses, whereas censoring was particularly consequential for antimony. Detailed reagent- and batch-level clinical-chemistry records were also unavailable. Furthermore, the inherited imputation models may not be fully compatible with some subsequent outcome specifications, including the additional albumin-adjusted analyses [33]. Although FDR correction was applied separately within five testing families, these procedures do not provide a single 5% FDR guarantee across all 380 linear comparisons, complementary analyses, or sensitivities, nor do they address cumulative multiplicity across SPES publications. Because the analyses reuse participants, assays, and imputation resources, independent replication remains necessary.

Future longitudinal studies incorporating standardized specimen collection, detailed inflammatory and exposure information, and paired renal-function measurements could help distinguish altered clearance and shared determinants from potential element-related effects. For selenium, concurrent measurements of total and ionized calcium and protein fractions would help clarify the observed biochemical relationships, while more detailed assessment of iron status would inform the interpretation of the cobalt findings.

## 4. Materials and Methods

### 4.1. Study Design and Participants

This secondary cross-sectional analysis used SPES data collected in Campania in 2016–2017. Potential participants were selected from municipal registries within the predefined environmental geostratification framework, and clinical, questionnaire, dietary, and biospecimen information was collected at enrollment [13,14]. Exclusion of 17 recruits from outside the study area left 4188 participants in 174 municipalities grouped into 21 geographical clusters. The available parent cohort determined sample size; no outcome-specific recruitment or population sampling weights were used. Reporting follows STROBE and its Molecular Epidemiology extension [34,35]; the completed checklist is provided as Supplementary File S2.

Fully adjusted analyses required questionnaire-derived covariates, restricting the primary population to 4137 participants. For covariate validity, weight, height, and calculated body mass index (BMI) were required to be finite and within broad prespecified plausibility ranges (30–250 kg, 130–220 cm, and 12–70 kg/m^2^, respectively; inclusive bounds). Six records did not satisfy these data-validity criteria and were excluded from fully adjusted models, leaving a maximum of 4131 before element-specific assay restrictions. These checks concerned covariate data, not eligibility for enrollment in SPES. Each model also required a positive finite outcome and eligible exposure. The shared serum measurements and imputation resource have been used in geographical, dietary, tumor-history, and lipid/blood-count analyses [15,16,17,36]. The present outcome families, eligibility criteria, and model specifications differed from those reports. In particular, this analysis did not impose the disease-history restriction used in the lipid/blood-count paper. Its contribution is the renal, hepatic, electrolyte/mineral, and protein/storage assessment, not new recruitment or assays (Supplementary Methods SM1).

### 4.2. Biospecimen Collection and Laboratory Measurements

Participants were instructed to fast for at least 8 h, and fasting status was checked before venipuncture. Fresh specimens were allocated to routine biochemical and virological testing within 3 h of collection. Separate serum aliquots were processed for biomonitoring. Blood collected in BD SST II Advance tubes was barcode-linked and transported under temperature monitoring; serum for biobanking was separated at 2000× *g* for 10 min at 4 °C, aliquoted, and stored at −80 °C. These were distinct fresh clinical-testing and frozen biomonitoring pathways. Collection and serum analytical procedures have been described previously [14,16].

Routine biochemical testing was performed in an accredited clinical laboratory. A contemporaneous SPES laboratory report specifies the Jaffé method for creatinine and International Federation of Clinical Chemistry (IFCC) methods at 37 °C for ALT and GGT. Additional recorded assay methods are described in Supplementary Methods SM2.1.

At the Department of Chemistry of the Istituto Zooprofilattico Sperimentale del Mezzogiorno (IZSM), elements were measured by inductively coupled plasma mass spectrometry (ICP-MS) using a NexION 350 ICP-MS (PerkinElmer, Shelton, CT, USA) after 1:10 dilution of 500 µL serum with nitric-acid diluent. Daily five-point calibration, online Y-89/Rh-103/Lu-175 internal standards, reagent blanks, internal controls, and certified reference materials were used; duplicate measurements were averaged [16]. Nineteen elements were analyzed, with Fe-56 representing iron and Fe-57 retained only for concordance. Concentrations were recorded in µg/L except iron and zinc in mg/L. Isotope identifiers and complete analytical details are provided in Supplementary Methods SM2 and Table S1a,b. Arsenic and mercury were measured as total elements without chemical speciation; specific inorganic and organic species cannot be inferred retrospectively from these measurements. This study involved no new laboratory measurements.

### 4.3. Outcomes and Analytical Hierarchy

The four primary outcomes were serum creatinine (mg/dL), ALT (U/L), GGT (U/L), and potassium (mmol/L), representing filtration-related renal physiology, hepatocellular biochemical activity, hepatobiliary/metabolic enzyme activity, and electrolyte regulation. ALT and GGT were not treated as direct measurements of hepatic synthetic function. Secondary outcomes comprised urea and uric acid; AST, alkaline phosphatase, total and direct bilirubin, and pseudocholinesterase; and sodium, chloride, total calcium, and phosphate. Albumin, total protein, beta-2 and gamma globulin fractions, and ferritin formed an exploratory protein transport/storage family. Globulin values were electrophoretic-fraction concentrations. Table S9 specifies all units and source fields. Outcomes were analyzed on the natural-log scale after validation of finite positive values. The 2021 race-free Chronic Kidney Disease Epidemiology Collaboration (CKD-EPI) creatinine equation was used only for descriptive eGFR translation and filtration-restricted sensitivities, not as a separately modeled primary endpoint or a diagnostic classification [37].

### 4.4. Missing Values, Left-Censoring, and Exposure Scales

Primary models reused the 30 completed datasets from the existing SPES multiple-imputation procedure (40 iterations; seed 20260111) [15,38]. The parent imputations were not regenerated for this revision. The four primary and most secondary outcomes were observed measurements. Four protein-related outcomes each had one missing original value and were completed by predictive mean matching. Element concentrations below the LOQ were handled by element-specific censored models rather than fixed substitution [39]. Assay unavailability was distinct from left-censoring. Unavailable mercury and nickel assays were not imputed, although available below-LOQ observations remained eligible for completion. The parent high-concentration plausibility rule set flagged values to missing before eligible imputation; unflagged quantified values were retained. Detailed imputation procedures, assay-state definitions, and thresholds are provided in Supplementary Methods SM2 and Table S1a,b.

For each element, the log-concentration median and IQR were calculated within each completed reference dataset; the medians of the 30 corresponding constants were fixed across outcomes and continuous sensitivities. Reference populations included 4137 participants for most elements and the available-assay subsets for mercury and nickel. Models used z = [ln(concentration) − c]/s, with fixed center c and log-IQR s. The original percentage estimate was 100[exp(β) − 1]. For main-text presentation, the estimate and log-scale confidence limits were algebraically expressed per doubling using 100{exp[β ln(2)/s] − 1}. This re-expression leaves *p* values, q values, sample sizes, and model assumptions unchanged. A doubling is a common relative increment, not a common absolute dose. The transformation is detailed in Supplementary Methods SM3; Tables S1b and S2a–d retain the scale constants and original primary log-IQR estimates.

### 4.5. Covariate Adjustment and Statistical Inference

Model A included the element, age spline, sex, and the 21-level geographical cluster factor. Primary Model B additionally included BMI spline, smoking, alcohol intake, physical activity, socioeconomic deprivation, potential occupational exposure, and total energy intake. Alcohol, activity, and energy were transformed as log(1 + value); unknown questionnaire responses were retained as explicit categories. Age knots were 21, 30, 40, and 48 years, and BMI knots were 20.37, 24.62, and 31.14 kg/m^2^. Definitions and coding are reported in Table S10.

The geographical fixed effects adjusted for between-cluster differences, while the bias-reduced CR2 cluster-robust covariance accounted for residual correlation among residents of the same municipality, with a small-sample adjustment [40]. The 21 fixed-effect groups were distinct from the 174 municipalities used for covariance clustering. Coefficients and covariance estimates were obtained within each completed dataset and pooled using Rubin’s rules and Barnard–Rubin degrees of freedom; global spline tests used multivariate pooling [41,42,43]. Benjamini–Hochberg adjustment controlled the FDR at 5% across all 76 primary comparisons [44]. The renal/metabolic, hepatic, electrolyte/mineral, and exploratory protein/storage families were adjusted separately across 38, 95, 76, and 95 comparisons. Confidence intervals are model-based 95% intervals, not simultaneous multiplicity-adjusted intervals. q values denote Benjamini–Hochberg-adjusted *p* values. Correction used all *p* values in each family, not only nominally significant comparisons. These separate procedures do not provide one 5% FDR guarantee for the combined 380 tests or for analyses across publications.

### 4.6. Complementary Models and Sensitivity Analyses

Restricted cubic splines with three fixed knots at the 10th, 50th, and 90th percentiles of log element concentration assessed global associations and departures from linearity in separate 76-test families [45]. Another family contained 76 exposure-by-sex interactions. Eight elements with below-LOQ proportions under 10% among available assays—lithium, arsenic, cobalt, copper, iron, selenium, strontium, and zinc—were also modeled simultaneously, yielding 32 conditional coefficients across the primary outcomes. Joint mixture estimates used fixed quartile scores of 0–3. Component coefficients were summed and their full covariance matrix was used to estimate the variance of a simultaneous one-category contrast [46]. Each eight-, 12-, or 17-element set was corrected separately across its four outcomes. Fixed-quantile derivation, support criteria, and set composition are specified in Supplementary Methods SM3 and SM4.

Sensitivity analyses examined alternative censoring models, outcome-inclusive completion, quantified-only measurements, quantification status, assay-availability weighting, influential observations, outcome resolution, geographic and covariance specifications, filtration and clinical restrictions, selected nutrients, and calendar time. An unclustered HC1 covariance analysis changed the within-municipality dependence assumption as well as the finite-sample adjustment; it was not treated as an additional primary family. Outcome-inclusive completion examined compatibility with the substantive model [33]. The final specification and conditional execution criteria preceded the run of 22 August 2026 but followed preliminary examination of the data and computational procedures; the analysis was not prospectively registered before data collection. The original specifications, eligibility differences, and sensitivity identifiers are in Supplementary Methods SM5 and Table S6. Sensitivity estimates retain their original correction sets and are not independent replications.

Filtration-restricted Sensitivity Analysis 8 separately applied approximate CKD-EPI 2021 eGFR thresholds of ≥60 and ≥90 mL/min/1.73 m^2^ to nonrenal outcomes; Sensitivity Analysis 9 added natural-log creatinine to those models. Creatinine, urea, and uric acid were excluded from both sensitivity grids. Creatinine and creatinine-derived eGFR were not included as covariates in the primary creatinine model.

In additional post hoc exploratory analyses undertaken during revision, the selenium–total-calcium and selenium–total-protein models were refitted after adding natural-log albumin as a continuous covariate to Model B. These two additions retained the same 4131 participants, 30 completed datasets, fixed selenium scaling, geographical adjustment, and municipality-clustered CR2 inference. Rubin pooling used Barnard–Rubin degrees of freedom with complete-data degrees of freedom G − 1 = 173. The coefficient reduction was summarized descriptively on the common log scale, not as mediation or variance explained. Two-sided nominal *p* values and pointwise 95% confidence intervals were reported without changing the original FDR families (Supplementary Methods SM5.1; Table S13).

### 4.7. Ethics, Software, and Research Materials

The parent SPES programme was conducted in accordance with the Declaration of Helsinki and approved by the Ethics Committee of IRCCS Fondazione G. Pascale (Extraordinary Commissioner’s Resolution No. 590, 3 August 2016). The present secondary analysis used de-identified data under the parent study’s data-governance framework and involved no new participant contact, specimen collection, or laboratory testing. The original analyses were implemented in R 4.5.1; the additional albumin-adjusted analyses were implemented in R 4.6.1. The R analysis and reporting source code, model specifications, and technical documentation are available from Zenodo (DOI: 10.5281/zenodo.23314158) [47]. Supplementary Methods SM6 describes the public code release and the controlled-access inputs required for reproduction. Participant-level records remain under institutional access controls. Generative-AI tools assisted with manuscript preparation, literature searching, and generation and review of analysis code; their use is disclosed in the Acknowledgments.

## 5. Conclusions

This secondary cross-sectional study identified small primary associations whose biological and clinical relevance remains uncertain. Lithium–creatinine was compatible with renal-handling differences, while antimony associations were sensitive to low-concentration handling and remained uncertain. Secondary and exploratory findings require independent confirmation. Albumin adjustment approximately halved the selenium coefficients for total calcium and total protein, supporting an interpretation centered on overlapping serum biochemistry rather than independent physiological effects. The results do not establish causality, clinical toxicity, mediation, or an indication to alter trace-element intake.

## Figures and Tables

**Table 1:** Demographic and selected biochemical characteristics of participants with questionnaire data.

Characteristic	Observed *n*	Summary
Age, years	4137	34.9 (8.8), mean (SD)
Women	4137	2236 (54.0%)
Creatinine, mg/dL	4137	0.80 (0.70–0.90)
ALT, U/L	4137	35 (30–40)
GGT, U/L	4137	19 (14–28)
Potassium, mmol/L	4137	4.6 (4.4–4.8)
Urea, mg/dL	4137	33 (28–39)
Uric acid, mg/dL	4137	4.7 (3.9–5.7)
Total bilirubin, mg/dL	4137	0.60 (0.50–0.80)
Direct bilirubin, mg/dL	4137	0.30 (0.20–0.30)
Ferritin, ng/mL	4137	56.0 (22.6–134.6)
Albumin, g/dL	4136	4.29 (4.08–4.49)
Total protein, g/dL	4136	7.10 (6.90–7.50)
Total calcium, mg/dL	4137	9.1 (8.8–9.4)
Phosphate, mg/dL	4137	3.9 (3.5–4.3)

Values are median (interquartile range) unless otherwise stated. Demographic and laboratory summaries use the existing 4137-person questionnaire population, not the smaller element-specific regression populations. Albumin and total protein summaries use the 4136 original observed values; their single missing values were imputed for eligible association models. SD, standard deviation; ALT, alanine aminotransferase; GGT, gamma-glutamyl transferase.

**Table 2:** Serum-element distributions and quantification among participants with questionnaire data (*N* = 4137).

Element	Unit	Available Assays, *n*	LOQ	Quantified Median (Q1–Q3)	Below LOQ, *n* (%)
Lithium	µg/L	4137	0.0048	1.0268 (0.73903–1.4915)	24 (0.6%)
Beryllium	µg/L	4137	0.0015	0.0528 (0.018975–0.097025)	2873 (69.4%)
Cadmium	µg/L	4137	0.001	0.0504 (0.01915–0.1408)	1850 (44.7%)
Arsenic	µg/L	4136	0.0015	1.6976 (0.7512–3.6263)	295 (7.1%)
Chromium	µg/L	4134	0.0015	0.7084 (0.3563–1.4569)	1691 (40.9%)
Cobalt	µg/L	4134	0.0016	0.9439 (0.7152–1.2176)	33 (0.8%)
Copper	µg/L	4134	0.3007	989.33 (841.79–1164.6)	1 (<0.1%)
Iron	mg/L	4134	0.0007	1.2141 (0.90605–1.5812)	8 (0.2%)
Mercury	µg/L	3353	0.1192	1.6471 (0.76935–3.2155)	866 (25.8%)
Manganese	µg/L	4136	0.0096	1.2027 (0.78533–1.68)	414 (10.0%)
Molybdenum	µg/L	4137	0.0099	0.9101 (0.5724–1.2855)	617 (14.9%)
Nickel	µg/L	2277	0.0212	1.9416 (0.95185–3.6572)	687 (30.2%)
Lead	µg/L	4137	0.0182	0.3006 (0.1103–0.70045)	2934 (70.9%)
Antimony	µg/L	4137	0.002	0.16555 (0.1096–0.23758)	1506 (36.4%)
Selenium	µg/L	4137	0.1114	196.12 (161.99–232.15)	1 (<0.1%)
Strontium	µg/L	4137	0.0193	28.31 (22.65–35.582)	1 (<0.1%)
Thallium	µg/L	4137	0.0012	0.0316 (0.0177–0.0578)	1063 (25.7%)
Vanadium	µg/L	4134	0.0087	0.4763 (0.2957–0.74803)	469 (11.3%)
Zinc	mg/L	3957	0.0004	1.4295 (1.2147–1.6581)	1 (<0.1%)

Concentration summaries describe unflagged directly quantified measurements, not the distribution after completion. Below-LOQ percentages use available assays; nonzero percentages below 0.1% are shown as <0.1%. Available assays can also include high-concentration plausibility-flagged observations; full state counts remain in Table S1a. Unavailable assays are distinct from below-LOQ measurements. Iron is represented by Fe-56; Fe-57 was used only for concordance. LOQ, limit of quantification; Q1–Q3, first–third quartiles.

**Table 3:** Primary associations meeting the FDR criterion across all 76 comparisons.

Association	*n*	Difference per Doubling, % (95% CI)	*p*	q
Lithium–creatinine	4131	+0.54 (+0.22 to +0.86)	0.001	0.028
Antimony–ALT	4131	+0.60 (+0.27 to +0.94)	<0.001	0.020
Antimony–potassium	4131	+0.17 (+0.10 to +0.24)	<0.001	<0.001

All differences correspond to a doubling of the named serum element. Model B adjusts for age, sex, body mass index (BMI), smoking, alcohol intake, physical activity, deprivation, occupational exposure, energy intake, and 21 geographical fixed-effect groups. Confidence intervals use bias-reduced municipality-clustered CR2 covariance and multiple-imputation pooling. q values are Benjamini–Hochberg-adjusted *p* values across all 76 primary comparisons; the 95% intervals are not simultaneous multiplicity-adjusted intervals. Original fixed log-IQR estimates are in Table S2a–d. For lithium, that increment corresponds to a 2.02-fold concentration ratio, so the two scales nearly coincide. ALT, alanine aminotransferase; FDR, false discovery rate; CI, confidence interval; IQR, interquartile range.

**Table 4:** Secondary and exploratory associations meeting their original within-family FDR criteria.

Testing Family	Biochemical Outcome	Serum Element	Difference per Doubling, % (95% CI)	q
Renal/metabolic	Uric acid	Lithium	+1.35 (+0.81 to +1.90)	<0.001
Renal/metabolic	Uric acid	Arsenic	+0.50 (+0.30 to +0.70)	<0.001
Renal/metabolic	Uric acid	Selenium	+1.76 (+0.59 to +2.94)	0.031
Renal/metabolic	Uric acid	Strontium	+4.24 (+2.70 to +5.82)	<0.001
Hepatic	Alkaline phosphatase	Copper	+3.39 (+1.36 to +5.46)	0.018
Hepatic	Alkaline phosphatase	Vanadium	−0.67 (−1.06 to −0.29)	0.015
Hepatic	Total bilirubin	Copper	−5.31 (−8.26 to −2.27)	0.015
Hepatic	Total bilirubin	Iron	+10.01 (+4.24 to +16.10)	0.015
Hepatic	Direct bilirubin	Copper	−5.06 (−7.80 to −2.24)	0.015
Hepatic	Direct bilirubin	Iron	+9.23 (+3.97 to +14.76)	0.015
Electrolyte/mineral	Sodium	Thallium	−0.03 (−0.05 to −0.01)	0.020
Electrolyte/mineral	Chloride	Beryllium	+0.06 (+0.02 to +0.09)	0.008
Electrolyte/mineral	Chloride	Antimony	+0.09 (+0.06 to +0.12)	<0.001
Electrolyte/mineral	Chloride	Selenium	−0.22 (−0.38 to −0.07)	0.046
Electrolyte/mineral	Chloride	Vanadium	−0.06 (−0.11 to −0.02)	0.039
Electrolyte/mineral	Total calcium	Selenium	+0.61 (+0.38 to +0.83)	<0.001
Electrolyte/mineral	Phosphate	Cobalt	−0.55 (−0.82 to −0.28)	0.002
Electrolyte/mineral	Phosphate	Iron	−1.55 (−2.02 to −1.08)	<0.001
Electrolyte/mineral	Phosphate	Zinc	−1.70 (−2.58 to −0.82)	0.003
Protein/storage (exploratory)	Albumin	Selenium	+1.29 (+0.82 to +1.76)	<0.001
Protein/storage (exploratory)	Total protein	Selenium	+1.37 (+0.88 to +1.86)	<0.001
Protein/storage (exploratory)	Ferritin	Cobalt	−6.05 (−8.72 to −3.30)	0.001
Protein/storage (exploratory)	Ferritin	Iron	+17.25 (+6.59 to +28.99)	0.028

## Data Availability

The principal and supporting results are reported in this article and its Supplementary Materials. The R analysis and reporting source code, model specifications, and technical documentation are available from Zenodo (DOI: 10.5281/zenodo.23314158) [47]. Participant-level data and completed datasets contain health and biomonitoring information and are subject to ethical, privacy, and institutional governance restrictions. Requests for de-identified participant-level data may be considered by the Istituto Zooprofilattico Sperimentale del Mezzogiorno, subject to institutional authorization and applicable data-use arrangements. Participant-level reproduction requires the controlled-access inputs and the existing 30-completion imputation object. The public code includes synthetic tests but no saved participant-level estimates or aggregate result datasets. Reproduction of the study estimates requires authorized access to the underlying inputs.

## Supplementary Materials

The following supporting information can be downloaded at: https://www.mdpi.com/article/10.3390/ijms27209313/s1.

## Abbreviations

ALT, alanine aminotransferase; AST, aspartate aminotransferase; BMI, body mass index; CI, confidence interval; eGFR, estimated glomerular filtration rate; FDR, false discovery rate; GGT, gamma-glutamyl transferase; ICP-MS, inductively coupled plasma mass spectrometry; IQR, interquartile range; IZSM, Istituto Zooprofilattico Sperimentale del Mezzogiorno; LOQ, limit of quantification; SPES, Exposure Study on Susceptible People.
