# Supplementary material for: Serum Trace Elements and Concurrent Renal, Hepatic, and Electrolyte Biomarkers: A Secondary Cross-Sectional Analysis of SPES
Source: Int J Mol Sci. 2026 Oct 20;27(20):9313. doi: 10.3390/ijms27209313 (PMC13705075; doi:10.3390/ijms27209313)
Supplement: Supplementary file 1 [file ijms-27-09313-s001.zip › ijms-4625882_STROBE_STROBE_ME_Checklist--File S2.pdf]

STROBE and STROBE-ME reporting checklist

Serum Trace Elements and Concurrent Renal, Hepatic, and Electrolyte Biomarkers: A Secondary Cross-Sectional Analysis of SPES

Reporting checklist for the revised main manuscript (20 pages) and Supplementary Material (29 pages). Page numbers refer to the accompanying clean PDF versions; section and table identifiers provide stable locators.

Item descriptions are abbreviated from the cross-sectional STROBE checklist and the STROBE-ME extension. Locations and qualifications are reported for each applicable item.

A. STROBE: cross-sectional study

Item	Reporting focus	Location	Coverage / qualification
1a	Study design in title/abstract	Title and Abstract, p. 1	Reported: secondary cross-sectional analysis.
1b	Balanced abstract	Abstract, p. 1	Reported: design, methods, small primary magnitude, antimony uncertainty, secondary findings, and attenuation after albumin adjustment.
2	Scientific background and rationale	Introduction, pp. 2–3	Reported: biochemical rationale, selected human evidence, SPES setting.
3	Objectives and prespecified hypotheses	Introduction, pp. 2–3; Methods 4.3 and 4.6, pp. 14–16; SM1, pp. 1–2	Reported with qualification: no directional pair-specific hypotheses; final original specification followed preliminary data examination. Additional albumin models are identified as additional post hoc exploratory analyses.
4	Key design elements	Methods 4.1, pp. 13–14; Abstract, p. 1	Reported: secondary cross-sectional design.
5	Setting, recruitment, dates	Methods 4.1–4.2, pp. 13–14; SM1–SM2, pp. 1–4	Reported: Campania registry recruitment framework and 2016–2017 collection; parent methods cited.
6a	Participant eligibility and selection	Methods 4.1, pp. 13–14; SM1, pp. 1–2; Figure S1, p. 28	Participant selection and covariate data-validity checks are distinguished; detailed parent recruitment criteria are in cited parent publications.
7	Outcomes, exposures and covariates	Methods 4.2–4.5, pp. 14–15; Table 2, pp. 4–5; Tables S1a–S1b and S9–S10, pp. 11–12, 24–25	Reported; biochemical measurements are not disease diagnoses.
8	Data sources and measurement	Methods 4.2–4.4, pp. 14–15; SM2, pp. 2–4	Accredited routine-testing pathway, report-level assay principles and ICP-MS methods are described; historical reagent/batch and calibration-traceability records remain limited.
9	Potential sources of bias	Methods 4.4–4.6, pp. 14–16; Discussion, pp. 10–13; SM5, pp. 6–8	Reported: censoring, assay subsets, geographical adjustment, sensitivities, residual confounding, reverse causation, and limits of preanalytical information. Large sample size is not presented as removal of systematic bias.
10	How study size was determined	Methods 4.1, pp. 13–14	Reported: available parent cohort; no new outcome-specific recruitment.
11	Handling quantitative variables	Methods 4.3–4.6, pp. 14–16; SM3–SM5.1, pp. 4–8; Table S10, p. 25	Reported: log outcomes, fixed element scaling, doubling display, spline knots and transformations.
12a	Statistical methods and confounding	Methods 4.5–4.6, pp. 15–16; SM4–SM5.1, pp. 5–8	Reported: covariates, CR2 inference, pooling and separate multiplicity families.
12b	Subgroups and interactions	Methods 4.6, pp. 15–16; Results 2.5, p. 9; Tables S5a–S5d, pp. 18–20	Reported: exposure-by-sex tests and specified restriction sensitivities.
12c	Missing data	Methods 4.1 and 4.4, pp. 13–15; SM1–SM2, pp. 1–4; Tables S1a–S1b and S9, pp. 11–12, 24	Reported: questionnaire restriction, missing versus censored assays and eligible imputation.
12d	Analytical treatment of sampling strategy	Methods 4.1 and 4.5, pp. 13–15	Reported: no population sampling weights; geographical fixed effects and municipality covariance. Registry sampling does not establish representativeness.
12e	Sensitivity analyses	Methods 4.6, pp. 15–16; Results 2.4, pp. 8–9; SM5–SM5.1, pp. 6–8; Tables S6 and S13, pp. 21, 28	The original sensitivity specifications and selected estimates are reported. Two additional post hoc albumin-adjusted analyses are documented separately in SM5.1 and Table S13.
13a	Numbers at study stages	Results 2.1, p. 3; Figure S1, p. 28; Tables 1–2, pp. 4–5; Tables S1a–S2d, pp. 11–16	Reported from enrollment through analysis; six covariate data-validity exclusions are distinguished from enrollment criteria. Invitation-stage denominators are not supplied.

Item	Reporting focus	Location	Coverage / qualification
13b	Exclusion and nonparticipation	Results 2.1, p. 3; Methods 4.1, pp. 13–14; Figure S1, p. 28	Partial: post-enrollment exclusions and assay restrictions reported; pre-enrollment refusal/nonparticipation reasons not reconstructed.
13c	Participant-flow diagram	Figure S1, p. 28	Reported.
14a	Descriptive participant characteristics	Table 1, p. 4; Results 2.1, p. 3; Table S10, p. 25	Partial: age, sex and selected biochemistry reported. Table S10 defines adjustment variables but does not provide their full cohort distributions.
14b	Missing data by variable	Table 1, p. 4; Tables S1a–S1b and S9, pp. 11–12, 24; SM1–SM2, pp. 1–4	Observed biochemical denominators and assay states reported; full covariate missing/unknown frequencies are not tabulated.
15	Outcome summaries	Table 1, p. 4; Results 2.1, p. 3	Selected observed-data outcome summaries and their denominators are reported for the cross-sectional study.
16a	Estimates, precision and adjustment	Results 2.2–2.5, pp. 5–9; Tables 3–4, pp. 5, 7–8; Tables S2a–S2d and S11–S13, pp. 13–16, 26–28	Reported with qualification: Model B adjusted estimates and pointwise 95% intervals are presented. Complete Model A and wholly unadjusted estimates are not tabulated. The two additional albumin-adjusted sensitivities report nominal p values.
16b	Category boundaries	Methods 4.6, pp. 15–16; SM3–SM5, pp. 4–7; Tables S1a–S1b and S10, pp. 11–12, 25	Reported: fixed quantile derivation, quartile category scores and covariate coding.
16c	Absolute-risk translations, where relevant	SM3, pp. 4–5; Tables S3a–S3c, p. 17	Not applicable to event risk. Existing original-unit biochemical predictions are labelled descriptive/extrapolative, not absolute disease risks.
17	Other analyses	Results 2.4–2.5, pp. 8–9; SR2–SR5, pp. 9–10; Tables S3a–S3c, S5a–S5d, S8a–S8c, S11–S13	Reported with the original analytical hierarchy and multiplicity scope. New albumin sensitivities are separately labeled; coefficient attenuation is not interpreted as mediation.
18	Key results in relation to objectives	Discussion, pp. 10–13; Conclusions, p. 17	Reported: magnitude, uncertainty, primary/secondary hierarchy, and overlapping selenium–protein/calcium observations are distinguished.
19	Limitations and direction of uncertainty	Discussion, pp. 10–13; SM1–SM5.1, pp. 1–8	Reported: temporal ambiguity, confounding, preanalytical and matrix/speciation limitations, censoring sensitivity, and imputation compatibility. Bias magnitudes are not estimated.
20	Interpretation, multiplicity and other evidence	Discussion, pp. 10–13; Table S7, p. 22	Reported: matrix-specific human comparisons, related outcomes, separate inferential families, and cumulative multiplicity across SPES reports.
21	Generalizability	Discussion, p. 12	Reported: young geographically structured cohort, questionnaire restriction and non-equivalence to companion cohorts.
22	Funding and funder role	Funding statement, p. 17	Funding and funder-role statement reported.

B. STROBE-ME: molecular epidemiology extension

Item	Reporting focus	Location	Coverage / qualification
ME-1	Identify biomarkers in title/abstract	Title and Abstract, p. 1	Reported: serum elements and concurrent biochemical outcomes.
ME-2	Biological rationale	Introduction, pp. 2–3; Discussion, pp. 10–13	Reported with selected human evidence.
ME-3	A priori biomarker hypotheses or expected values	Introduction, pp. 2–3; Methods 4.6, pp. 15–16; SM1, pp. 1–2	Exploratory direction/magnitude objective; no retrospective pair-specific hypotheses or expected concentrations claimed.
ME-4	Biomarker-specific design features	Methods 4.1–4.4, pp. 13–15; SM1–SM3, pp. 1–5	Secondary use of concurrent specimens; not a nested case-control or case-cohort design.
ME-4.1	Specimen collection conditions	Methods 4.2, p. 14; SM2.1, pp. 2–3	Reported: fasting, specimen allocation, serum tubes, transport and analytical aliquot. Exact participant-level fasting durations are not claimed.
ME-4.2	Specimen processing	Methods 4.2, p. 14; SM2.1, pp. 2–3	Reported: fresh-testing pathway versus refrigerated centrifugation and frozen aliquots.
ME-4.3	Storage and handling	Methods 4.2, p. 14; SM2.1, pp. 2–3	Reported: −80 °C storage and thawing/mixing. Complete specimen-specific storage and freeze–thaw histories are not provided.
ME-4.4	New biomarkers and physicochemical properties	Methods 4.2–4.3, p. 14	Not a validation study of a newly developed biomarker; established measurements are used.
ME-6	Participant factors affecting biomarkers	Methods 4.1–4.2 and 4.5, pp. 13–15; Discussion, p. 12; Table S10, p. 25	Reported: fasting procedure, clinical/questionnaire covariates and residual confounding. A uniformly disease-free cohort is not asserted.
ME-8	Assays, calibration, thresholds and outliers	Methods 4.2 and 4.4, pp. 14–15; SM2–SM3, pp. 2–5; Table 2, pp. 4–5; Tables S1a–S1b, pp. 11–12	Report-level routine-assay principles and ICP-MS/LOQ/plausibility methods are reported. Historical calibration traceability and some reagent/batch details are unavailable.
ME-12	Biomarker statistical treatment	Methods 4.3–4.6, pp. 14–16; SM2–SM5.1, pp. 2–8	Reported: transformations, censoring, missingness, scaling and inferential methods.
ME-12.1	Validity and reliability of biomarker measurement	SM2.1, pp. 2–3; Discussion, pp. 12–13	Partial: reported routine-assay principles and the ICP-MS duplicate/calibration/control framework are described. No new laboratory validation or comprehensive numerical reliability audit is claimed.
ME-13	Loss of specimens or measurements	Results 2.1, p. 3; Table 2, pp. 4–5; Tables S1a–S1b, pp. 11–12; Figure S1, p. 28	Assay-state counts and model exclusions reported; availability flags do not establish laboratory reasons for unavailability.
ME-14.1	Biomarker distributions	Tables 1–2, pp. 4–5; Tables S1a–S1b, pp. 11–12	Partial: quantified-element medians and interquartile ranges, assay-state denominators, and exposure scaling are reported. Means, full observed ranges, and variances are not tabulated.
ME-19	Measurement limitations	Discussion, p. 13; SM2–SM5.1, pp. 2–8	Reported: matrix, speciation, assay subsets, censoring, and unavailable historical calibration/reagent/batch details.
ME-20	Biological interpretation	Discussion, pp. 10–13; Table S7, p. 22; Table S13, p. 28	Reported: directionality, shared determinants, human evidence and limits of treatment/experimental analogies.
ME-22.1	Consent, ethics and anonymity	Methods 4.7, pp. 16–17; ethics and consent statements, p. 17; data-access statement, p. 18; SM6, p. 8	Reported with qualification: parent ethics approval, written consent, de-identified secondary data use, and restricted access are documented. The identifiability classification of the original biological specimens is not explicitly stated in the supplied materials.

Sources: STROBE, von Elm et al. (2007), doi:10.1016/S0140-6736(07)61602-X; STROBE-ME, Gallo et al. (2011), doi:10.1371/journal.pmed.1001117. See main references 34–35 and https://www.strobe-statement.org/checklists/.
