# Supplementary material for: Serum Trace Elements and Concurrent Renal, Hepatic, and Electrolyte Biomarkers: A Secondary Cross-Sectional Analysis of SPES
Source: Int J Mol Sci. 2026 Oct 20;27(20):9313. doi: 10.3390/ijms27209313 (PMC13705075; doi:10.3390/ijms27209313)
Supplement: Supplementary file 1 [file ijms-27-09313-s001.zip › ijms-4625882_Supplementary_Material--File S1.pdf]

MDPI

International Journal of Molecular Sciences

Supplementary Materials

Serum Trace Elements and Concurrent Renal, Hepatic, and Electrolyte Biomarkers: A Secondary Cross-Sectional Analysis of SPES

Correspondence: Carlo Buonerba; carbuone@hotmail.com.

Contents: detailed methods SM1–SM6; supplementary results SR1–SR5; Tables S1–S13; Figures S1–S2. Main-text continuous estimates are per doubling; original supplementary tables retain the fixed element-specific log-IQR unless explicitly labelled otherwise.

References use the numbering of the main manuscript. These materials provide detailed methods, supplementary results, complete primary tables, and selected sensitivity comparisons. Outcome and covariate definitions are provided in Tables S9–S10, and sensitivity-analysis identifiers are defined in Table S6.

Supplementary Methods

SM1. Study Population, Data Sources, and Analytical Scope

The SPES parent study combined environmental characterization, population recruitment, clinical examination, lifestyle and dietary questionnaires, and venous sampling at enrollment [13–16]. The analytical cohort comprised 4,188 participants after exclusion of 17 recruits from outside the study area. The primary analysis population comprised the 4,137 participants with questionnaire data. Fifty-one participants without questionnaire data were excluded from imputation-model fitting and fully adjusted association models. The existing imputation procedure allowed prediction of eligible laboratory values for these participants, permitting their inclusion in the minimally adjusted full-cohort analysis (Sensitivity Analysis 17). A complete questionnaire history was not imputed for inclusion in Model B.

Prespecified covariate data-validity checks required finite weight (30–250 kg), height (130–220 cm), and calculated BMI (12–70 kg/m^2), with inclusive bounds. Six records failed these checks and were excluded from fully adjusted models (maximum n = 4,131); these were analytical, not enrollment, exclusions. Each outcome–element pair additionally required a finite positive outcome and an eligible exposure value. Mercury and nickel analyses were restricted to available assays; primary mercury models included 3,350 participants and nickel models 2,276. Tables S2a–S2d report the sample size for each model. The 21 municipal-cluster groups included as fixed effects were distinct from the 174 municipalities used for covariance clustering.

Biochemical and questionnaire variables were obtained from the SPES analytical dataset. Existing multiple imputations supplied eligible element concentrations and four protein-related outcomes; the remaining biochemical outcomes were observed values. Participants and serum measurements overlap with published geographical, dietary, tumor-history, and lipid/blood-count analyses, but the current analysis used distinct outcome families and eligibility rules [15–17,36]. Table S9 defines every outcome, and Table S10 defines the covariates. This analysis did not apply the disease-history restriction of the lipid/blood-count paper.

The present contribution is the renal, hepatic, electrolyte/mineral, and protein/storage outcome assessment; the reuse of participants, assays, and imputations does not provide independent replication.

The final secondary-analysis specification defined the exposure panel, outcome families, covariates, assay-state rules, and conditional sensitivity criteria before the run of 22 August 2026. Preliminary examination of the same data and computational procedures preceded that specification; the analysis was not prospectively registered before data collection. Huber regression was applied to primary associations meeting the FDR criterion, and recording-resolution analyses were applied to outcomes meeting the specified recording-grid criteria. These were additional evaluations of the same dataset rather than independent replication. The two additional albumin-adjusted selenium models were undertaken during peer review and are reported separately in SM5.1, SR5, and Table S13; they did not change the original testing hierarchy.

SM2. Laboratory Procedures, Assay States, and Imputation

SM2.1. Specimen collection, routine clinical chemistry, and serum ICP-MS

Participants were required to fast for at least 8 h, and fasting status was checked before venipuncture. Fresh specimens were allocated to biochemical and virological testing within 3 h of collection. Separate serum aliquots were prepared for biobanking. Blood collected in BD SST II Advance tubes (REF 366468; Becton Dickinson, Franklin Lakes, NJ, USA) was linked by barcode and transported under temperature monitoring. Serum for biobanking was separated by centrifugation at 2000 × g for 10 min at 4 °C, aliquoted into cryovials, and stored at −80 °C in a remotely monitored biobank. Before analysis, aliquots were brought to room temperature and mixed by gentle rotation [16].

Routine biochemical testing was performed in an accredited clinical laboratory. The available contemporaneous SPES report specifies a Jaffé method for creatinine and International Federation of Clinical Chemistry (IFCC) methods at 37 °C for ALT, GGT, AST, and alkaline phosphatase. Sodium and potassium are labelled ICT in the report; an electrode configuration is not specified. These descriptions concern the fresh clinical-testing pathway, not the ICP-MS trace-element panel.

The same report lists urease/GLDH for urea, enzymatic colorimetry for uric acid, Walters/Gerarde for bilirubin fractions, butyrylthiocholine at 37 °C for pseudocholinesterase, OCP colorimetry for total calcium, ultraviolet molybdate for phosphate, colorimetry for chloride, and chemiluminescence for ferritin. It labels an albumin assay as BCP and also reports an electrophoretic albumin fraction; beta-2 and gamma globulin concentrations are reported in the Capillarys (Sebia) electrophoresis panel. Total protein is listed without a separate assay principle. These report-level method designations do not establish uniform analyser, reagent, or calibration conditions across every study batch.

Specimen-level hemolysis indices and a C-reactive protein (CRP) measure for adjustment were not available in the analytical inputs. The present analysis did not account directly for menstrual status, menstrual blood loss, or supplement type and dose. Sex and dietary adjustment, high-concentration plausibility rules,

and medication-related sensitivities do not substitute for these factors. Their unmeasured contribution to potassium, iron, phosphate, bilirubin, ferritin, and cobalt-related associations could not be quantified.

The serum trace-element panel was measured at the IZSM Department of Chemistry using a PerkinElmer NexION 350 ICP-MS system (Waltham, MA, USA), equipped with a Meinhard concentric nebulizer (Golden, CO, USA), cyclonic spray chamber, quartz torch, and quartz injector with a 2-mm internal diameter. High-purity argon and helium were supplied by Rivoira (Milan, Italy). Preparation used 500 μL serum diluted 1:10 with nitric-acid diluent; the recorded reagent was superpure nitric acid (VWR, Leuven, Belgium). These procedures applied to the serum panel, separately from erythrocyte-chromium and specialist-substudy analyses [16].

Five calibration points were prepared daily for each element over an appropriate interval within the nominal 0.1–100 μg/L range in 0.5% nitric acid. PerkinElmer multielement and internal-standard stocks (Norwalk, CT, USA) were used. Y-89, Rh-103, and Lu-175 were introduced online; calibration linearity met $R^2 > 0.99$. Reagent blanks were monitored, variation across 20 blanks informed LOQ estimation, and internal controls and certified reference materials were used to assess accuracy and precision. The mean of duplicate measurements was retained [16]. Table S1b reports the numerical LOQs in the original channel units, including the concordance-only Fe-57 channel. Arsenic and mercury concentrations were total-element measurements without chemical speciation.

SM2.2. Assay availability, left-censoring, and high-concentration plausibility flags

An available assay was distinguished from an unavailable assay, and an available below-LOQ measurement was classified as left-censored. Table S1a gives available and unavailable counts and partitions available assays into quantified, left-censored, plausibility-flagged, and other missing observations. Every element had 4,137 records for participants with questionnaire data. The fixed-scaling reference population included eligible completed values and could exceed the number with an available assay; for zinc, reference N was 4,137 and available n was 3,957. The counts and distributions describe measurements from the parent SPES resource used in earlier reports [15–17] and are reproduced to describe the populations contributing to the present models.

The high-concentration plausibility criterion flagged measurements strictly greater than 20 times the element-specific upper reference limit adopted during parent preprocessing. It was not a specimen hemolysis index, batch-failure indicator, or clinical toxicity threshold. Flagged values were set to missing before eligible imputation; the original configuration permitted their imputation. Unflagged quantified values were retained. A sensitivity analysis excluded participants with flagged concentrations, while alternative censoring-completion analyses excluded them before fixing their eligible populations.

Unavailable assays could be completed only when their channel-specific proportion was ≤5% and the other imputation criteria were met. Unavailable mercury and nickel assays were not completed; available below-LOQ mercury and nickel values remained eligible for censoring-aware completion. High-concentration flags and other eligible missing values were handled under the parent specification. The assay-availability indicator does not record the operational reason for

an unavailable assay. Inverse-probability weighting examined sensitivity to selection conditional on measured predictors, under its stated assumptions.

SM2.3. Distributional families and numerical bounds

The parent chained-equations procedure used 30 completed datasets, 40 iterations, and seed 20260111. Model fitting, donor selection, and predictor selection were restricted to the 4,137 participants with questionnaire data. Gaussian Tobit models on log concentration were used for beryllium, cadmium, arsenic, chromium, mercury, nickel, lead, strontium, thallium, and vanadium. Gaussian Tobit models on Box–Cox scales were used for iron-56 (λ = 0.47), manganese (0.33), selenium (0.46), and zinc (0.70). Interval-censored Gamma models were used for lithium, cobalt, copper, iron-57, molybdenum, and antimony. Eligible non-censored missing observations were completed by predictive mean matching on the applicable working scale [15,38].

Age, sex, environmental-impact stratum, and geographical cluster were included in each conditional model. Additional predictors were retained using a deterministic target-specific procedure to limit categorical expansion and rank deficiency. Questionnaire skip states were coded explicitly rather than imputed as observed answers. The upper numerical censoring boundary was $LOQ^* = LOQ - \min[\max(10^{-9}, 10^{-6} \times |LOQ|), 0.5 \times LOQ]$, with a positivity floor of 10^{-12} in the channel's original unit. These numerical constraints are distinct from analytical LOQs. Table S1b reports the analytical thresholds; the formula above defines the distinct numerical censoring boundaries.

Predictive mean matching used five donors for 13 laboratory variables: albumin, total protein, beta-2 globulin, gamma globulin, hematocrit, hemoglobin, MCV, RDW, basophils, eosinophils, lymphocytes, neutrophils, and platelets. Only the first four were outcomes here, each with one missing original value among participants with questionnaire data. The four primary outcomes and remaining secondary outcomes used observed values. An observed-outcome sensitivity restricted the four protein outcomes to original measurements. Hematological variables contributed to the inherited imputation procedure but were not outcomes of this study. The existing completed datasets were reused in the additional albumin-adjusted analyses; no parent imputation was regenerated.

SM3. Fixed Exposure Scales and Predictions in Original Outcome Units

For each completed dataset m and element e, the median c_{em} and IQR s_{em} of log concentration were calculated in the fixed reference population. The center c_e and scale s_e were the medians of the 30 dataset-specific constants, calculated separately rather than from stacked datasets. The standardized exposure $z_e = [\ln(x_e) - c_e]/s_e$ was applied unchanged across outcomes, subgroups, and continuous sensitivity analyses. Reference populations comprised participants with questionnaire data for most elements and the corresponding available-assay subsets for mercury and nickel. Reference percentiles were calculated within each completed dataset, then fixed at the median of each corresponding percentile across the 30 datasets. The 10th, 50th, and 90th log-concentration percentiles defined exposure-spline knots; the 25th, 50th, and 75th percentiles defined mixture categories. Table S1b reports scale constants and concentration ratios.

For a coefficient β on the fixed log-IQR scale s, $100[\exp(\beta) - 1]$ gives the percentage difference per original log-IQR; the corresponding concentration ratio is

exp(s). The per-doubling estimate is 100{exp[β ln(2)/s] − 1}, with the same transformation applied to both pooled log-scale confidence limits. Main-text continuous estimates and Figure 1 use this per-doubling expression. Tables S2a–S2d, S3a–S3c, S5c, S8b–S8c, S11, and S12 retain the original fixed log-IQR scale. Binary quantification-status contrasts and mixture-category estimates are not rescaled. The transformation does not change p values, q values, model sample sizes, or the original testing families. Table S13 reports the additional selenium models per doubling.

Absolute-outcome predictions were calculated by setting z = 0 and z = 1 for every participant while retaining individual covariate values. Predicted log outcomes were exponentiated, and predictions and differences were averaged within each completed dataset and then across datasets. No residual-smearing correction was applied; the resulting summaries are averages of conditional geometric-mean predictions rather than arithmetic conditional means. The exposure settings were exp(c) and exp(c + s), not the first and third quartiles. The between-completion SD quantifies variability of the contrast across completed datasets and is not a total SE, CI, or pooled inferential interval.

The lithium prediction settings were 1.024 and 2.065 μg/L. The antimony settings were 0.1018 and 14.185 μg/L. The latter upper value exceeded the central concentration distribution and the 10-μg/L parent plausibility cutoff. Consequently, the antimony original-unit predictions are extrapolations of the fitted relationship rather than examples of typical observed exposure differences. Table S3a retains these derived quantities and separates them from the primary coefficients, confidence intervals, and inferential results. Neither the reference-plus-log-IQR prediction nor the per-doubling display should be interpreted as a within-person intervention effect.

For the descriptive lithium eGFR calculation, the 2021 Chronic Kidney Disease Epidemiology Collaboration (CKD-EPI) creatinine equation was applied to each participant's predicted creatinine, age, and sex at both exposure settings before averaging [37]. The resulting difference was −0.663 mL/min/1.73 m^2. Calibration traceability of the historical creatinine measurements to isotope-dilution mass spectrometry could not be retrospectively verified. The eGFR difference was a transformation of the modeled creatinine contrast, not a separately estimated renal endpoint, measured filtration value, or diagnostic classification. In Sensitivity Analysis 8, the same equation was applied to observed creatinine to define the ≥60 and ≥90 mL/min/1.73 m^2 restrictions for nonrenal outcomes. Creatinine, urea, and uric acid were not included in these nonrenal restriction grids; Sensitivity Analysis 9 likewise concerned only nonrenal outcomes. Creatinine-derived eGFR was not used as an independent filtration adjustment in a model with creatinine as outcome.

SM4. Regression, Pooling, Multiplicity, and Mixtures

Primary Model B comprised the scaled element; age and BMI restricted cubic splines; sex; smoking; log(1 + alcohol), log(1 + physical activity), and log(1 + energy intake); the recorded deprivation score; potential occupational exposure; and 21 municipal-cluster fixed effects. To account for residual correlation among participants residing in the same municipality, primary standard errors used the bias-reduced CR2 cluster-robust estimator with a small-sample adjustment. The HC1 sensitivity used conventional heteroskedasticity-robust standard errors

without municipality clustering and therefore changed the geographic covariance assumption as well as the finite-sample correction [40].

Within each completion, the coefficient estimate and its CR2 covariance were retained. Scalar pooling used the average coefficient $\bar{Q}$, average within-completion variance $\bar{U}$, between-completion variance B, and total variance $T = \bar{U} + (1 + 1/M)B$, with M = 30. Barnard–Rubin small-sample degrees of freedom used the complete-data municipality count minus one, along with the missing-information adjustment [41,42]. Exposure-response splines used three fixed knots at the reference 10th, 50th, and 90th percentiles of log concentration. The global association test jointly evaluated the linear and nonlinear basis coefficients, using CR2-based multivariate pooling [43,45]; the nonlinear-component test evaluated departure from linearity separately.

The Benjamini–Hochberg procedure controlled the false discovery rate at 5% separately within the primary 76-test family, the four additional biochemical families (38/95/76/95 comparisons), 76 global spline tests, 76 nonlinear-component tests, 76 sex interactions, and 32 coefficients from the principal eight-element high-support co-exposure model [44]. Each of the three mixture sets constituted a separate four-outcome family. All q values were calculated using their complete prespecified families, including results not displayed in selected tables. The primary, secondary, and exploratory designations were retained throughout interpretation. These are separate within-family corrections, not one 5% FDR guarantee across all 380 linear comparisons, complementary/sensitivity analyses, or cumulative SPES publications. The hierarchy was not a prospectively registered confirmatory framework, and borderline findings remained exploratory.

The analytical-support classification used the proportion of available assays reported below the LOQ. Mercury and nickel were classified separately as assay-limited, and iron-57 was reserved for concordance. Among the remaining elements, a below-LOQ proportion <10% defined the eight-element low-censoring set; proportions from 10% through 30% defined the additional intermediate group; and proportions >30% defined the remaining model-dependent group. These categories describe quantification support, not analytical accuracy or toxicological priority. The principal eight-element set comprised lithium, arsenic, cobalt, copper, iron-56, selenium, strontium, and zinc. The 12-element set added manganese, molybdenum, thallium, and vanadium; the 17-element set additionally included beryllium, cadmium, chromium, lead, and antimony. Mercury, nickel, and iron-57 were excluded from all sets. Fixed 25th, 50th, and 75th percentile cut-points defined category scores of 0–3. Within each completed dataset, the joint coefficient was the sum of component coefficients, with variance $1'V1$ from their full covariance matrix; estimates were then pooled [46]. The contrast represents a simultaneous one-quartile-category increase across all included elements. Tables S1a and S5d identify the analytical-support classes and mixture sets.

SM5. Sensitivity Analysis Specifications

Alternative censoring completion used outcome–element-specific populations satisfying assay-availability, censoring-bound, covariate, and plausibility criteria. Quantified concentrations, observed outcomes, covariates, and population membership were held fixed across 60 repeated completions. The outcome-inclusive alternative added the concurrent log outcome for beryllium, cadmium, chro-

mium, nickel, lead, and antimony across the four primary outcomes. This comparison examined compatibility between the imputation and substantive models [33]. Both alternatives retained the primary fixed scaling but could differ from the primary analysis in eligibility and concentration representation. Original identifiers for these and all other sensitivities are given in Table S6.

Quantification-status models compared quantified with below-LOQ observations among eligible available assays. Eligibility required at least 50 observations in each group, 30 municipalities overall, and 10 municipalities represented in each group. Lithium had only 24 below-LOQ records and failed the minimum group-size criterion; its comparison was therefore not fitted. Quantified-only continuous models included only unflagged quantified concentrations and retained the original fixed log-IQR. These models concern a narrower concentration range and a different eligible population from the completed-data analysis.

For mercury and nickel, assay availability was modeled by logistic regression using primary-model covariates, within-cluster-centered visit day, and the concurrent log outcome. Stabilized weights used the eligible population's marginal availability probability as numerator and the participant-specific predicted probability as denominator. Weights for available assays were truncated at the first and 99th percentiles. The weighted association model also included centered visit day. Interpretation requires conditional exchangeability and positivity given the measured predictors; visit day was a calendar-time variable rather than a reconstructed analytical-batch identifier.

Outcome trimming applied fixed observed first and 99th percentiles. Huber robust regression was restricted to the three FDR-supported primary associations and supplied point estimates only. Filtration restrictions (Sensitivity Analysis 8: ≥60 and ≥90 mL/min/1.73 m^2) and additional log-creatinine adjustment (Sensitivity Analysis 9) concerned nonrenal outcomes only; creatinine, urea, and uric acid were excluded from both grids. Other specifications included outcome-specific condition exclusions, a medication-free restriction, restriction to observed protein outcomes, a municipality random-intercept model, unclustered HC1 covariance, and omission of deprivation. These analyses evaluated different eligibility or model assumptions, with model-specific populations and correction sets retained in the result tables.

Additional nutrient adjustment included protein intake in g/day for creatinine, urea, and uric acid; dietary calcium in mg/day for calcium and phosphate; and dietary iron in mg/day for ferritin. The existing output contains 114 additional nutrient-adjusted fits and 266 unchanged primary-model records. No nutrient term was added to ALT or potassium. The minimally adjusted full-cohort analysis included the 51 participants without questionnaire data and changed both the population and adjustment specification. Iron-57 concordance assessed agreement with iron-56 rather than a second independent iron exposure. Exclusion of high-concentration flags evaluated sensitivity to imputation after the parent plausibility rule.

The potassium contextual assessment used the existing primary iron-56, zinc, manganese, copper, lead, and cadmium results to examine a possible generalized erythrocyte-release pattern. No independent specimen-integrity measurement was added. The calendar-time analysis added within-cluster-centered visit day

among participants with questionnaire data. Outcome-resolution analyses excluded recorded minima or used rank-inverse-normal and grouped-value outcomes when the recording-distribution criteria were met. Only minimum-exclusion models retained the percentage-outcome scale; rank and grouped models had different estimands. Their directions are summarized separately in Table S3c.

SM5.1. Additional post hoc albumin-adjusted selenium analyses

The original selenium models for total calcium, albumin, and total protein were first reproduced. Two additional exploratory specifications added natural-log albumin as one continuous term to the original Model B for total calcium and total protein separately. Albumin was not adjusted for itself. All five models used identical eligible participant sets (n = 4,131), 174 municipalities, 21 geographical fixed-effect groups, and the existing 30 completed datasets. The original age and BMI spline terms, covariate coding, natural-log outcomes, fixed selenium center and log-IQR, and municipality-clustered CR2 covariance were retained. No additional imputation or outcome transformation was introduced.

Within-completion coefficients and CR2 variances were pooled using Rubin's rules and Barnard–Rubin degrees of freedom with complete-data degrees of freedom $G - 1 = 173$ [40–42]. Per-doubling percentages and confidence limits used the transformation in SM3. Coefficient attenuation was calculated as $100 \times [1 - \beta(\text{albumin-adjusted})/\beta(\text{original})]$ on the common log scale. This was descriptive, not a mediated proportion, proportion of variance explained, or formal paired test of coefficient change. The two additional models report nominal two-sided p values and pointwise 95% confidence intervals, without a new FDR claim.

The outcome remained measured total calcium; no formula-corrected or ionized-calcium outcome was analyzed. Ionized calcium was not available in these analytical inputs. No non-albumin protein outcome, mediation analysis, interaction, or new element screen was undertaken. The inherited imputation resource was retained, so compatibility with every conditional model was not guaranteed [33]. The analyses were run in R 4.6.1; the code and recorded software environment accompany the reproducibility materials.

SM6. Research Materials and Access

Tables S2a–S2d report all 76 primary associations. Table S4 defines the four secondary and exploratory families, and main Table 4 reports all 23 associations meeting their original within-family criteria. Tables S5a–S5d and S8a–S12 provide complementary and sensitivity results, with the original exposure contrasts, populations, confidence intervals, and testing families retained. The additional albumin-adjusted estimates are reported separately in Table S13.

The R analysis and reporting source code, model specifications, input dictionary, software information, and synthetic tests are available from Zenodo (DOI: 10.5281/zenodo.23314158) [47]. The source includes the two additional albumin-adjusted selenium analyses. No participant-level workbooks, completed imputation objects, model estimates, or aggregate result tables are included in the public code archive.

The public repository enables inspection of the analysis workflow and execution of synthetic tests without SPES participant data. Empirical model fitting requires the controlled-access participant inputs and the existing 30 completed im-

putation datasets; the public code does not download or distribute these resources. Any outputs generated by an authorized local run remain outside the public deposit. Access to participant-level inputs is governed by the main Data Availability Statement.

Supplementary Results

SR1. Complete Association Families and Relevant Non-Findings

Tables S2a–S2d report the complete primary results on the original fixed log-IQR scale, with sample size, municipality count, nominal p values, and primary CR2 and alternative HC1 q values. Three associations met the primary 76-test criterion. Under HC1, ALT–vanadium and GGT–iron also met the threshold; they were not treated as additional primary findings. For GGT, lead ($p = 0.388$; $q = 0.608$) and cadmium ($p = 0.557$; $q = 0.756$) did not show nominal support. Copper ($p = 0.040$; $q = 0.276$), iron ($p = 0.015$; $q = 0.146$), and selenium ($p = 0.022$; $q = 0.185$) had positive nominal associations, and vanadium had an inverse nominal association ($p = 0.006$; $q = 0.093$); none met the primary FDR criterion. These inferential results are unchanged by per-doubling presentation. No urea, AST, or pseudocholinesterase association met its respective secondary-family criterion. Further complementary and sensitivity results are reported in Tables S5a–S5d and S8a–S12.

Twenty-three secondary/exploratory associations met their separately applied family criteria: four renal/metabolic, six hepatic, nine electrolyte/mineral, and four protein transport/storage. Zinc point estimates for total and direct bilirubin were positive, but did not meet the hepatic-family criterion, providing only partial correspondence with the external iron/copper/zinc study [11]. No beta-2 or gamma globulin result met the exploratory-family criterion. The manuscript's biological discussion combines related observations without changing these original statistical families. The small selenium–chloride finding ($q = 0.046$) was treated as hypothesis-generating, without a substantive mechanistic interpretation.

SR2. Primary Sensitivity Results and Exposure Representation

The lithium–creatinine association remained positive across the examined specifications. The original fixed-log-IQR estimate was +0.54%, compared with +0.57% under alternative completion, +0.70% among quantified measurements, and +0.43% with Huber regression. The corresponding per-doubling expressions are +0.54%, +0.56%, +0.69%, and +0.42%. Differences between the two display scales within any one specification reflect algebraic rescaling, not a change to that model. Table S11 retains the original-scale estimates and correction sets, and Figure S2 separates continuous from quantification-status comparisons.

For antimony, alternative completion reduced the original ALT estimate from +4.39% to +2.31% and potassium from +1.22% to +0.64%. Quantified-only continuous estimates were negative and imprecise, whereas quantified-versus-below-LOQ comparisons were positive. These results do not establish a biological threshold at the LOQ: the binary and continuous contrasts answer different questions and the restricted analysis has a different population and concentration range. Adding the outcome to the alternative completion model yielded similar estimates to the outcome-omitting completion. This does not identify which aspect of the primary-versus-alternative specifications accounts for the difference.

https://doi.org/10.3390/ijms27209313

Under the municipality random-intercept specification, the ALT estimate remained positive but did not meet its original sensitivity-family FDR criterion (q = 0.199); potassium retained support (q = 0.019). The corresponding estimates and intervals are in Table S11. In the existing nonrenal log-creatinine-adjusted sensitivity, the per-doubling antimony estimates were +0.61% for ALT (95% CI, +0.28% to +0.94%) and +0.17% for potassium (+0.10% to +0.24%), both with n = 4,131; these did not resolve the uncertainty arising from low-concentration representation.

SR3. Secondary Sensitivity Context

Table S12 compares the main secondary estimates with alternative-completion and quantified-only results for all 23 secondary or exploratory pairs meeting their original family criteria. Continuous estimates use the fixed element-specific log-IQR scale. Iron–ferritin, selenium–albumin, and strontium–uric-acid estimates were similar under main and alternative completion. Antimony–chloride attenuated from +0.62% to +0.32%; its quantified-only estimate was −0.52% (95% CI, −0.99% to −0.04%; nominal p = 0.032; q = 0.188; n = 2,622). Its interval excluded zero, but the comparison did not meet the original quantified-only sensitivity-family FDR criterion. Beryllium–chloride estimates were approximately +0.19%, +0.08%, and +0.07% across the three specifications; thallium–sodium estimates were −0.16%, −0.14%, and +0.10%. Each sensitivity retained its eligible population and original multiplicity set.

These comparisons describe sensitivity of the existing estimates, not independent confirmation or a new correction family. Quantified-only restrictions can materially reduce exposure range, while alternative completion also applies assay and plausibility eligibility criteria. The four protein outcomes each had one imputed original measurement; the observed-outcome sensitivity retained their original measured data. The nutrient-adjustment output includes unchanged records for outcomes to which no nutrient was added, so those duplicates cannot be interpreted as new evidence of robustness.

SR4. Complementary Models and Derived Quantities

Global spline significance and evidence of nonlinearity are distinct. Seven global associations met their separate criterion, but only selenium–creatinine met the nonlinear-component criterion. The available global tests alone do not establish a U-shaped, J-shaped, or threshold relationship. The mercury–ALT sex interaction is a ratio of exposure-associated outcome ratios between men and women, not a sex difference in ALT itself; Table S5b retains its original log-IQR contrast. No coefficient in the 32-test mutually adjusted family met the criterion, whereas the principal mixture–creatinine estimate met its separate four-outcome criterion. The 12- and 17-element sets retained their own correction families.

Original-unit predictions in Table S3a are averages of exponentiated conditional log-outcome predictions, not arithmetic conditional means. The lithium-derived eGFR difference is a transformation of the same modeled creatinine contrast. For antimony, the upper prediction setting lies outside the central distribution and above the parent plausibility cutoff. The reported between-completion SD describes variability among completed datasets, not total inferential uncertainty. Outcome-resolution analyses are reported separately in Table S3c and do not make these extrapolative antimony contrasts typical observed differences.

SR5. Albumin-adjusted selenium associations

In additional post hoc analyses, adding natural-log albumin to Model B attenuated the selenium association with total calcium from +0.61% per doubling (95% CI, +0.38% to +0.83%) to +0.29% (+0.08% to +0.50%; nominal p = 0.008). The corresponding total-protein estimate decreased from +1.37% (+0.88% to +1.86%) to +0.65% (+0.32% to +0.99%; nominal p = 0.00014). The log-scale selenium coefficients were reduced by 53.1% and 52.1%, respectively. All specifications retained the same 4,131 participants and 30 completed datasets (Table S13).

Substantial attenuation was the principal finding; the residual positive conditional associations did not demonstrate a direct selenium effect or mediation. Albumin and total protein remain overlapping measurements.

Supplementary Tables

Table S1a. Assay availability and quantification among 4,137 participants with questionnaire data.

Channel	Available / unavailable n	Quantified n	Below LOQ n	Below LOQ / available	Flagged n	Support class
Li-7	4,137 / 0	4,110	24	0.6%	3	Strict high
Be-9	4,137 / 0	1,264	2,873	69.4%	0	Extended/model-dependent
Cd-111	4,137 / 0	2,287	1,850	44.7%	0	Extended/model-dependent
As-75	4,136 / 1	3,841	295	7.1%	0	Strict high
Cr-52	4,134 / 3	2,443	1,691	40.9%	0	Extended/model-dependent
Co-59	4,134 / 3	4,101	33	0.8%	0	Strict high
Cu-63	4,134 / 3	4,133	1	0.0%	0	Strict high
Fe-56	4,134 / 3	4,126	8	0.2%	0	Strict high
Hg-202	3,353 / 784	2,482	866	25.8%	5	Assay-limited
Mn-55	4,136 / 1	3,722	414	10.0%	0	Common/intermediate
Mo-98	4,137 / 0	3,517	617	14.9%	3	Common/intermediate
Ni-60	2,277 / 1,860	1,584	687	30.2%	6	Assay-limited
Pb-208	4,137 / 0	1,199	2,934	70.9%	4	Extended/model-dependent
Sb-121	4,137 / 0	2,626	1,506	36.4%	5	Extended/model-dependent
Se-78	4,137 / 0	4,136	1	0.0%	0	Strict high
Sr-88	4,137 / 0	4,136	1	0.0%	0	Strict high
Tl-205	4,137 / 0	3,073	1,063	25.7%	1	Common/intermediate
V-51	4,134 / 3	3,656	469	11.3%	9	Common/intermediate
Zn-66	3,957 / 180	3,956	1	0.0%	0	Strict high; 180 raw flag-0 records (4.35%) met the prespecified ≤5% completion-eligibility threshold

Flag 1 denotes an available assay and flag 0 an unavailable assay. Within available assays, quantified, below-LOQ, high-concentration plausibility-flagged, and other missing observations sum to the available count. Other available-assay missing counts were zero for all 19 channels. The additional Fe-57 channel was used only for concordance and is reported in Table S1b. Below-LOQ percentages use available assays as their denominator. Availability flags do not specify the cause of unavailability. The high-concentration plausibility criterion is unrelated to hemolysis. Support classes describe quantification and eligibility, not analytical accuracy or toxicological categories; numerical criteria are in SM4. LOQ, limit of quantification.

Table S1b. Channel-specific LOQs, observed quantified concentration summaries, and fixed exposure scales.

Channel	Unit	LOQ	Quantified median (Q1–Q3)	Fixed log-IQR	Concentration ratio exp(s)	Plausibility cutoff
Li-7	µg/L	0.0048	1.0268 (0.73903–1.4915)	0.70147	2.017	300
Be-9	µg/L	0.0015	0.0528 (0.018975–0.097025)	2.41725	11.215	2
Cd-111	µg/L	0.001	0.0504 (0.01915–0.1408)	4.68484	108.293	10.9
As-75	µg/L	0.0015	1.6976 (0.7512–3.6263)	1.71064	5.532	153.3
Cr-52	µg/L	0.0015	0.7084 (0.3563–1.4569)	6.83873	933.307	76.8
Co-59	µg/L	0.0016	0.9439 (0.7152–1.2176)	0.53423	1.706	17.6
Cu-63	µg/L	0.3007	989.33 (841.79–1164.6)	0.32487	1.384	33360
Fe-56	mg/L	0.0007	1.2141 (0.90605–1.5812)	0.55938	1.750	43.54
Fe-57	mg/L	0.0006	1.2045 (0.8927–1.5774)	0.57069	1.769	43.54
Hg-202	µg/L	0.1192	1.6471 (0.76935–3.2155)	3.06386	21.410	44.5
Mn-55	µg/L	0.0096	1.2027 (0.78533–1.68)	0.96868	2.634	71.2
Mo-98	µg/L	0.0099	0.9101 (0.5724–1.2855)	1.29964	3.668	33.4
Ni-60	µg/L	0.0212	1.9416 (0.95185–3.6572)	4.91280	136.019	48.3
Pb-208	µg/L	0.0182	0.3006 (0.1103–0.70045)	1.62530	5.080	24.8
Sb-121	µg/L	0.002	0.16555 (0.1096–0.23758)	4.93691	139.340	10
Se-78	µg/L	0.1114	196.12 (161.99–232.15)	0.35987	1.433	1670
Sr-88	µg/L	0.0193	28.31 (22.65–35.582)	0.45181	1.571	920
Tl-205	µg/L	0.0012	0.0316 (0.0177–0.0578)	3.65798	38.783	1.4
V-51	µg/L	0.0087	0.4763 (0.2957–0.74803)	1.17745	3.246	3.2
Zn-66	mg/L	0.0004	1.4295 (1.2147–1.6581)	0.31691	1.373	32.5

Quantified concentration summaries were calculated from unflagged quantified observations. Fixed log-IQR scales are the medians of 30 reference-population log-IQRs calculated separately across completed datasets; their concentration ratios can therefore differ from ratios based on quantified-only quartiles. Fe-57 was used for concordance only. High-concentration plausibility cutoffs equal 20 times the adopted upper reference limits and are distinct from analytical LOQs. The formula in SM2.3 defines the computational LOQ* boundary. Reference N is 4,137 except for available-assay mercury (3,353) and nickel (2,277).

Table S2a. Complete primary associations for creatinine.

Channel	n	G	% difference (95% CI)	p	CR2 q	HC1 q
Li-7	4,131	174	+0.54 (+0.22 to +0.87)	0.001	0.028	0.013
Be-9	4,131	174	−0.01 (−0.50 to +0.48)	0.956	0.991	0.985
Cd-111	4,131	174	−0.10 (−0.85 to +0.66)	0.801	0.895	0.872
As-75	4,131	174	+0.11 (−0.19 to +0.41)	0.463	0.651	0.663
Cr-52	4,131	174	+0.78 (−0.01 to +1.57)	0.053	0.335	0.185
Co-59	4,131	174	−0.25 (−0.53 to +0.02)	0.072	0.382	0.142
Cu-63	4,131	174	−0.24 (−0.53 to +0.05)	0.108	0.433	0.345
Fe-56	4,131	174	−0.13 (−0.65 to +0.38)	0.606	0.779	0.644
Hg-202	3,350	172	+0.45 (−0.41 to +1.31)	0.309	0.574	0.558
Mn-55	4,131	174	−0.14 (−0.39 to +0.10)	0.252	0.574	0.345
Mo-98	4,131	174	+0.21 (−0.09 to +0.52)	0.169	0.504	0.309
Ni-60	2,276	168	+0.69 (−0.53 to +1.93)	0.265	0.574	0.503
Pb-208	4,131	174	−0.04 (−0.38 to +0.31)	0.832	0.904	0.909
Sb-121	4,131	174	−0.04 (−0.87 to +0.79)	0.924	0.975	0.955
Se-78	4,131	174	+0.21 (−0.20 to +0.62)	0.314	0.574	0.561
Sr-88	4,131	174	+0.43 (−0.05 to +0.91)	0.079	0.382	0.189
Tl-205	4,131	174	−0.38 (−1.09 to +0.34)	0.296	0.574	0.558
V-51	4,131	174	+0.02 (−0.37 to +0.41)	0.923	0.975	0.954
Zn-66	4,131	174	−0.05 (−0.37 to +0.28)	0.779	0.891	0.864

Model B; G, number of municipalities; fixed element-specific log-IQR scale; 30 completed datasets. CR2 q values were calculated across the complete 76-test primary family covering all four outcomes. HC1 q values were calculated separately for the unclustered covariance sensitivity analysis. CI, confidence interval; CR2, small-sample bias-reduced cluster-robust covariance; HC1, unclustered heteroskedasticity-consistent covariance.

Table S2b. Complete primary associations for alanine aminotransferase (ALT).

Channel	n	G	% difference (95% CI)	p	CR2 q	HC1 q
Li-7	4,131	174	+0.42 (−0.51 to +1.36)	0.374	0.608	0.598
Be-9	4,131	174	−0.54 (−1.78 to +0.72)	0.395	0.608	0.609
Cd-111	4,131	174	+1.07 (−0.95 to +3.12)	0.300	0.574	0.558
As-75	4,131	174	−0.38 (−1.12 to +0.36)	0.307	0.574	0.558
Cr-52	4,131	174	+1.56 (−0.51 to +3.67)	0.139	0.495	0.345
Co-59	4,131	174	−0.19 (−0.70 to +0.32)	0.459	0.651	0.702
Cu-63	4,131	174	−0.22 (−1.14 to +0.72)	0.648	0.794	0.735
Fe-56	4,131	174	+0.01 (−0.92 to +0.95)	0.984	0.991	0.991
Hg-202	3,350	172	−3.03 (−5.40 to −0.59)	0.015	0.146	0.076
Mn-55	4,131	174	−0.47 (−1.00 to +0.05)	0.077	0.382	0.197
Mo-98	4,131	174	+0.68 (−0.25 to +1.62)	0.151	0.498	0.189
Ni-60	2,276	168	+0.60 (−1.96 to +3.23)	0.647	0.794	0.801
Pb-208	4,131	174	+0.56 (−0.65 to +1.79)	0.362	0.608	0.558
Sb-121	4,131	174	+4.39 (+1.92 to +6.92)	<0.001	0.020	<0.001
Se-78	4,131	174	+0.50 (−0.52 to +1.54)	0.332	0.574	0.598
Sr-88	4,131	174	+0.63 (−0.50 to +1.78)	0.273	0.574	0.558
Tl-205	4,131	174	−1.62 (−3.92 to +0.72)	0.172	0.504	0.345
V-51	4,131	174	−1.24 (−2.10 to −0.38)	0.005	0.093	0.013
Zn-66	4,131	174	−0.25 (−1.18 to +0.68)	0.596	0.779	0.718

Model B; G, number of municipalities; fixed element-specific log-IQR scale; 30 completed datasets. CR2 q values were calculated across the complete 76-test primary family covering all four outcomes. HC1 q values were calculated separately for the unclustered covariance sensitivity analysis. CI, confidence interval; CR2, small-sample bias-reduced cluster-robust covariance; HC1, unclustered heteroskedasticity-consistent covariance.

https://doi.org/10.3390/ijms27209313

Table S2c. Complete primary associations for gamma-glutamyl transferase (GGT).

Channel	n	G	% difference (95% CI)	p	CR2 q	HC1 q
Li-7	4,131	174	−0.40 (−1.62 to +0.83)	0.516	0.713	0.706
Be-9	4,131	174	−0.98 (−2.93 to +1.00)	0.328	0.574	0.558
Cd-111	4,131	174	−0.74 (−3.17 to +1.76)	0.557	0.756	0.735
As-75	4,131	174	−0.24 (−1.33 to +0.85)	0.661	0.797	0.787
Cr-52	4,131	174	+1.11 (−1.68 to +3.99)	0.437	0.649	0.619
Co-59	4,131	174	−0.52 (−1.45 to +0.42)	0.277	0.574	0.558
Cu-63	4,131	174	+1.20 (+0.06 to +2.36)	0.040	0.276	0.189
Fe-56	4,131	174	+2.12 (+0.41 to +3.87)	0.015	0.146	0.013
Hg-202	3,350	172	−2.76 (−5.81 to +0.40)	0.085	0.382	0.345
Mn-55	4,131	174	−0.35 (−1.16 to +0.47)	0.400	0.608	0.609
Mo-98	4,131	174	+0.01 (−0.88 to +0.90)	0.991	0.991	0.991
Ni-60	2,276	168	−2.36 (−6.02 to +1.44)	0.219	0.574	0.558
Pb-208	4,131	174	+0.60 (−0.77 to +2.00)	0.388	0.608	0.617
Sb-121	4,131	174	−1.47 (−4.07 to +1.21)	0.278	0.574	0.558
Se-78	4,131	174	+1.63 (+0.24 to +3.04)	0.022	0.185	0.167
Sr-88	4,131	174	+0.46 (−1.32 to +2.27)	0.615	0.779	0.735
Tl-205	4,131	174	−1.32 (−3.70 to +1.12)	0.285	0.574	0.598
V-51	4,131	174	−1.65 (−2.81 to −0.48)	0.006	0.093	0.066
Zn-66	4,131	174	+0.70 (−0.53 to +1.94)	0.263	0.574	0.558

Model B; G, number of municipalities; fixed element-specific log-IQR scale; 30 completed datasets. CR2 q values were calculated across the complete 76-test primary family covering all four outcomes. HC1 q values were calculated separately for the unclustered covariance sensitivity analysis. CI, confidence interval; CR2, small-sample bias-reduced cluster-robust covariance; HC1, unclustered heteroskedasticity-consistent covariance.

Table S2d. Complete primary associations for potassium.

Channel	n	G	% difference (95% CI)	p	CR2 q	HC1 q
Li-7	4,131	174	+0.00 (−0.22 to +0.22)	0.969	0.991	0.985
Be-9	4,131	174	+0.36 (+0.09 to +0.63)	0.010	0.131	0.068
Cd-111	4,131	174	+0.11 (−0.32 to +0.54)	0.611	0.779	0.735
As-75	4,131	174	+0.07 (−0.11 to +0.26)	0.444	0.649	0.598
Cr-52	4,131	174	+0.07 (−0.41 to +0.54)	0.785	0.891	0.863
Co-59	4,131	174	−0.06 (−0.18 to +0.06)	0.322	0.574	0.609
Cu-63	4,131	174	−0.03 (−0.23 to +0.17)	0.778	0.891	0.867
Fe-56	4,131	174	+0.07 (−0.09 to +0.23)	0.399	0.608	0.701
Hg-202	3,350	172	+0.38 (−0.30 to +1.06)	0.272	0.574	0.369
Mn-55	4,131	174	−0.01 (−0.11 to +0.09)	0.825	0.904	0.884
Mo-98	4,131	174	+0.03 (−0.16 to +0.22)	0.734	0.871	0.778
Ni-60	2,276	168	+0.46 (−0.15 to +1.07)	0.141	0.495	0.345
Pb-208	4,131	174	−0.22 (−0.47 to +0.03)	0.084	0.382	0.189
Sb-121	4,131	174	+1.22 (+0.69 to +1.75)	<0.001	<0.001	<0.001
Se-78	4,131	174	−0.11 (−0.32 to +0.10)	0.296	0.574	0.558
Sr-88	4,131	174	+0.17 (−0.06 to +0.39)	0.143	0.495	0.345
Tl-205	4,131	174	+0.55 (+0.04 to +1.07)	0.034	0.257	0.091
V-51	4,131	174	−0.15 (−0.33 to +0.03)	0.096	0.404	0.189
Zn-66	4,131	174	−0.15 (−0.37 to +0.07)	0.170	0.504	0.382

Model B; G, number of municipalities; fixed element-specific log-IQR scale; 30 completed datasets. CR2 q values were calculated across the complete 76-test primary family covering all four outcomes. HC1 q values were calculated separately for the unclustered covariance sensitivity analysis. CI, confidence interval; CR2, small-sample bias-reduced cluster-robust covariance; HC1, unclustered heteroskedasticity-consistent covariance.

Table S3a. Predictions in original outcome units and corresponding exposure settings.

Association	Exposure settings: z = 0 → 1	Predicted reference	Predicted shifted	Native difference
Lithium–Creatinine	1.024 → 2.0651 μg/L	0.824155	0.828629	+0.00447 mg/dL
Antimony–Alanine aminotransferase	0.1018 → 14.185 μg/L	34.4719	35.9849	+1.51296 U/L
Antimony–Potassium	0.1018 → 14.185 μg/L	4.55227	4.60775	+0.05549 mmol/L

Predictions are covariate-standardized averages of exponentiated log-scale predictions without residual smearing. The exposure contrast is from the fixed log-median to that value plus one log-IQR, rather than from the first to the third quartile. The upper antimony setting of 14.185 μg/L exceeds the 10-μg/L high-concentration plausibility cutoff and the central exposure distribution; both antimony predictions are extrapolative. Between-completion SD quantifies between-dataset variability rather than a total SE or CI. The approximate lithium eGFR difference of −0.663 mL/min/1.73 m^2 is a descriptive transformation of the creatinine contrast. All original-unit prediction values in this table refer to the original reference-plus-log-IQR contrast, not to doubling.

Table S3b. Modeled contrasts relative to outcome dispersion, recording increments, and biological variation.

Association	Native difference / outcome SD	Native difference / outcome IQR	Recording-increment ratio	Relative effect / published CVI
Lithium–creatinine	3.3%	2.2%	0.045 × 0.10 mg/dL	≈12%; creatinine CVI 4.4%
Antimony–ALT*	11.2%	15.1%	1.51 × 1 U/L	≈47%; ALT CVI 9.3%
Antimony–potassium*	14.7%	13.9%	0.555 × 0.10 mmol/L	≈31%; potassium CVI 3.9%

*Antimony values derive from the extrapolative exposure settings in Table S3a and do not describe typical individual changes. CVI ratios equal the modeled relative difference divided by the cited within-person coefficient of variation [18–21]. Comparisons with SD, IQR, recording increments, and CVI describe different aspects of effect magnitude; they are not validated clinical thresholds and do not alter population-level inference. The ratios are descriptive comparisons, not minimum clinically important differences or clinical decision limits.

Table S3c. Outcome-resolution sensitivity results for the three primary pairs.

Association	Minimum-exclusion n	Minimum-exclusion % (95% CI)	q	Rank-inverse-normal outcome	Grouped-value direction
Lithium–Creatinine	4,122	+0.52 (+0.20 to +0.85)	0.035	Positive; q = 0.017	Positive
Antimony–Alanine aminotransferase	4,130	+4.29 (+1.87 to +6.77)	0.017	Positive; q = 0.008	Positive
Antimony–Potassium	4,130	+1.21 (+0.68 to +1.73)	<0.001	Positive; q = 0.011	Positive

Minimum-exclusion models retain the percentage-difference scale for log-transformed outcomes. Rank-inverse-normal and grouped-outcome models use different scales and are summarized here by direction. The different outcome scales preclude direct numerical comparison with percentage differences.

https://doi.org/10.3390/ijms27209313

Table S4. Secondary and exploratory outcome families and multiplicity criteria.

Family	Outcomes	Tests	Supported	Inferential status
Renal	Urea; Uric acid	38	4	Secondary
Hepatic	Aspartate aminotransferase (AST); Alkaline phosphatase; Total bilirubin; Direct bilirubin; Pseudocholinesterase	95	6	Secondary
Electrolyte/mineral	Sodium; Chloride; Calcium; Phosphate	76	9	Secondary
Protein transport/storage	Albumin; Total protein; Beta-2 globulin fraction; Gamma globulin fraction; Ferritin	95	4	Exploratory

Main Table 4 reports all 23 secondary or exploratory associations meeting their within-family FDR criteria. This table specifies the outcome sets and multiplicity denominators for all 304 comparisons, including those not meeting the criteria. Benjamini–Hochberg adjustment was applied separately within each family. The protein transport/storage family is exploratory. Urea is recorded under the source identifier azotemia. FDR, false discovery rate.

Table S5a. Global spline functions meeting their global-family FDR threshold.

Outcome	Element	n	Global p	Global q	Nonlinear-component q
Creatinine	Lithium	4,131	<0.001	0.011	0.280
Creatinine	Selenium	4,131	0.001	0.036	0.028
Alanine aminotransferase	Manganese	4,131	0.005	0.049	0.135
Alanine aminotransferase	Antimony	4,131	0.003	0.041	0.589
Gamma-glutamyl transferase	Iron (Fe-56)	4,131	0.002	0.036	0.135
Gamma-glutamyl transferase	Selenium	4,131	0.003	0.042	0.149
Potassium	Antimony	4,131	<0.001	0.006	0.589

Global association and nonlinear-component tests were adjusted in separate 76-test BH families. Only creatinine–selenium met the nonlinear-family criterion. A global spline association can be present without evidence of departure from linearity. The complete global and nonlinear results are reported in Table S5a.

Table S5b. FDR-supported exposure-by-sex interaction.

Outcome / element	n / municipalities	Interaction ratio (95% CI)	p	q
ALT / mercury	3,350 / 172	1.065 (1.029–1.103)	<0.001	0.035

The interaction estimate is the male:female ratio of per-log-IQR geometric mean ratios, with female as the reference category. It compares sex-specific associations and is not an estimate of the association within men alone. BH adjustment included all 76 interaction tests. The mercury estimate applies to participants with an available assay.

Table S5c. Mutually adjusted coefficients for the principal eight-element set (strict-8).

Outcome	Element	n	Adjusted % difference (95% CI)	p	q
Creatinine	Lithium	4,131	+0.50 (+0.15 to +0.85)	0.006	0.088
Creatinine	Arsenic	4,131	+0.04 (−0.26 to +0.34)	0.784	0.854
Creatinine	Cobalt	4,131	−0.28 (−0.53 to −0.02)	0.037	0.199
Creatinine	Copper	4,131	−0.40 (−0.74 to −0.05)	0.025	0.198
Creatinine	Iron (Fe-56)	4,131	−0.09 (−0.59 to +0.41)	0.723	0.826
Creatinine	Selenium	4,131	+0.37 (−0.14 to +0.88)	0.150	0.442
Creatinine	Strontium	4,131	+0.42 (−0.15 to +0.98)	0.146	0.442
Creatinine	Zinc	4,131	−0.14 (−0.63 to +0.35)	0.562	0.707
Alanine aminotransferase	Lithium	4,131	+0.34 (−0.66 to +1.34)	0.506	0.707
Alanine aminotransferase	Arsenic	4,131	−0.47 (−1.18 to +0.26)	0.205	0.480
Alanine aminotransferase	Cobalt	4,131	−0.26 (−0.85 to +0.34)	0.400	0.640
Alanine aminotransferase	Copper	4,131	−0.43 (−1.69 to +0.85)	0.510	0.707
Alanine aminotransferase	Iron (Fe-56)	4,131	+0.04 (−1.00 to +1.09)	0.938	0.938
Alanine aminotransferase	Selenium	4,131	+0.94 (−0.39 to +2.30)	0.166	0.442
Alanine aminotransferase	Strontium	4,131	+0.76 (−0.70 to +2.24)	0.308	0.599
Alanine aminotransferase	Zinc	4,131	−0.67 (−2.02 to +0.70)	0.336	0.599
Gamma-glutamyl transferase	Lithium	4,131	−0.65 (−1.98 to +0.70)	0.344	0.599
Gamma-glutamyl transferase	Arsenic	4,131	−0.34 (−1.45 to +0.78)	0.545	0.707
Gamma-glutamyl transferase	Cobalt	4,131	−1.33 (−2.40 to −0.24)	0.017	0.186
Gamma-glutamyl transferase	Copper	4,131	+0.91 (−0.52 to +2.36)	0.210	0.480
Gamma-glutamyl transferase	Iron (Fe-56)	4,131	+2.47 (+0.87 to +4.11)	0.003	0.085
Gamma-glutamyl transferase	Selenium	4,131	+1.50 (−0.35 to +3.39)	0.112	0.442
Gamma-glutamyl transferase	Strontium	4,131	−0.20 (−2.37 to +2.03)	0.861	0.888
Gamma-glutamyl transferase	Zinc	4,131	−0.49 (−2.18 to +1.24)	0.575	0.707
Potassium	Lithium	4,131	−0.05 (−0.31 to +0.21)	0.709	0.826
Potassium	Arsenic	4,131	+0.09 (−0.10 to +0.28)	0.356	0.599
Potassium	Cobalt	4,131	−0.07 (−0.21 to +0.06)	0.262	0.559
Potassium	Copper	4,131	+0.03 (−0.23 to +0.29)	0.801	0.854
Potassium	Iron (Fe-56)	4,131	+0.14 (−0.06 to +0.33)	0.162	0.442
Potassium	Selenium	4,131	−0.10 (−0.40 to +0.19)	0.488	0.707
Potassium	Strontium	4,131	+0.31 (+0.03 to +0.60)	0.033	0.199
Potassium	Zinc	4,131	−0.24 (−0.50 to +0.02)	0.070	0.318

The eight continuous elements were included together with Model B covariates. Each coefficient uses that element's fixed log-IQR. The 32 coefficients form a separate BH family; none has $q \leq 0.05$. Confidence limits shown here are algebraic transformations of the original pooled log-scale limits. Nominal p values are reported from the existing pooled model outputs; q values retain Benjamini–Hochberg adjustment across all 32 mutually adjusted coefficients.

Table S5d. All joint mixture estimates, with separate correction within each four-outcome set.

Set	Outcome	n	Joint % difference (95% CI)	p	Within-set q
Strict-8 (principal)	Creatinine	4,131	+0.79 (+0.19 to +1.39)	0.010	0.042
Strict-8 (principal)	Alanine aminotransferase	4,131	+0.33 (−1.32 to +2.01)	0.696	0.696
Strict-8 (principal)	Gamma-glutamyl transferase	4,131	+1.76 (−0.96 to +4.55)	0.206	0.412
Strict-8 (principal)	Potassium	4,131	+0.08 (−0.27 to +0.43)	0.665	0.696
Common-12	Creatinine	4,131	+0.70 (−0.12 to +1.53)	0.096	0.382
Common-12	Alanine aminotransferase	4,131	−0.25 (−2.34 to +1.88)	0.815	0.815
Common-12	Gamma-glutamyl transferase	4,131	−0.40 (−3.27 to +2.55)	0.786	0.815
Common-12	Potassium	4,131	+0.24 (−0.19 to +0.68)	0.268	0.536
Extended-17	Creatinine	4,131	+0.84 (−0.13 to +1.82)	0.089	0.358
Extended-17	Alanine aminotransferase	4,131	+1.00 (−1.39 to +3.44)	0.414	0.552
Extended-17	Gamma-glutamyl transferase	4,131	+0.40 (−2.90 to +3.82)	0.812	0.812
Extended-17	Potassium	4,131	+0.32 (−0.16 to +0.82)	0.191	0.383

Each joint estimate represents a simultaneous one-quartile-category increase across the included elements. Fixed concentration cutpoints define scores 0–3. Strict-8 comprises Li, As, Co, Cu, Fe-56, Se, Sr, and Zn; common-12 adds Mn, Mo, Tl, and V; extended-17 adds Be, Cd, Cr, Pb, and Sb. BH adjustment was performed within each four-outcome set rather than across a combined 12-test family. All models included 174 municipalities. Confidence limits were obtained by transforming the original log-scale limits.

Table S6. Specifications, populations, and interpretation of the 23 original sensitivity analyses.

Identifier	Analysis	Scope	Interpretation
SA1	Model A minimal adjustment	All primary and secondary pairs	Minimally adjusted comparator; Model B is the primary specification.
SA2	Quantification status	Elements with any <LOQ	Quantified versus <LOQ among assays coded available.
SA3	Directly quantified continuous	Elements with sufficient quantified support	Fixed log-IQR scale within the restricted quantified exposure range.
SA4	Alternative censoring repeated completions	All censored elements	Pair-specific sample meeting assay-state, censoring-bound, covariate, and Plausibility flag criteria; quantified values and participant membership fixed across 60 completions.
SA5	Hg/Ni assay-availability IPW	Hg and Ni	Inverse-probability weighting for assay availability, with calendar-time adjustment rather than measured batch effects.
SA6	Outcome P1–P99 trimming	All outcomes	Trimming at fixed observed-outcome percentiles.
SA7	Huber robust regression	Primary FDR-supported pairs	Direction and magnitude only.
SA8	Impaired-filtration exclusions	Nonrenal outcomes	Approximate eGFR <60 and <90 exclusions.
SA9	Creatinine adjustment	Nonrenal outcomes	Adds log creatinine.
SA10	Reported-condition exclusions	Domain-relevant pairs	Domain-relevant self-reported condition exclusions.
SA11	Medication-free restriction	All outcomes	Restriction based on any reported medication use, without drug-class stratification.
SA12	Observed-outcome components	PMM-target outcomes	Requires original observed outcome.
SA13	Alternative geography	Primary pairs	Municipality random intercept plus cluster fixed effects.
SA14	HC1 covariance and BH adjustment	All 76 primary pairs	Unclustered heteroskedasticity-consistent covariance, differing from the primary analysis in clustering and finite-sample correction.
SA15	No deprivation	All primary pairs	Sensitivity to exclusion of the deprivation score.
SA16	Outcome-domain nutrient adjustment	Six outcomes	Protein intake for renal outcomes, calcium intake for calcium/phosphate, and iron intake for ferritin. Remaining records retain Model B; comprehensive food-source adjustment was not performed.
SA17	Full-cohort Model A	Eligible outcomes/core covariates	Includes 51 nonresponders only in minimal model.
SA18	Fe-57 concordance	All outcome families	Comparison of direction and magnitude with Fe-56; not an independent exposure.
SA19	Outcome-inclusive censored completion	Be/Cd/Cr/Ni/Pb/Sb × 4 primary outcomes	Concurrent outcome included in the censoring model; eligible participant membership fixed across 60 completions.
SA20	Plausibility flag participant exclusion	Element-specific flagged rows	Sensitivity to imputation of flagged values.
SA21	Potassium pattern assessment	K × Fe/Zn/Mn/Cu/Pb/Cd	Indirect assessment based on existing associations, without specimen-level hemolysis validation.
SA22	Calendar-time sensitivity	Responder primary pairs; Hg/Ni selection models	Within-cluster-centered visit day.
SA23	Laboratory floor/heaping sensitivity	Flagged outcomes	Minimum-value exclusion, rank-inverse-normal transformation, and grouped-value models for flagged outcomes.

Sensitivity Analysis 14 results are reported in the HC1 columns of Tables S2a–S2d. Sensitivity Analysis 8 separately applies the ≥60 and ≥90 mL/min/1.73 m^2 filtration restrictions. Sensitivity Analysis 23 includes minimum-exclusion, rank, and grouped-outcome models (Table S3c). Sensitivity Analysis 16 contains 114 distinct nutrient-adjusted fits and 266 unchanged Model B results. Sensitivity Analysis 7 reports point estimates only. The two additional albumin-adjusted selenium models are additional exploratory analyses documented in SM5.1 and Table S13; they were not added retrospectively to the original 23-analysis specification.

https://doi.org/10.3390/ijms27209313

Table S7. Selected human evidence used to interpret the primary and secondary findings.

Association or question	Human evidence	Comparison and limitation
Lithium–creatinine	Enderle et al.: 928 adults; plasma lithium inversely associated with eGFR, chiefly below about 75 mL/min/1.73 m² [3].	The comparator modeled lithium rather than creatinine and involved an older population and plasma measurements. Directional agreement supports consideration of renal handling, not a causal effect or a direct clearance measurement in the present study.
Uric acid	Xu et al.: 3,957 adults; plasma arsenic and strontium associated with incident hyperuricemia [22]. NHANES: +0.119 mg/dL uric acid per doubling of blood selenium (95% CI 0.020–0.218) [23].	Longitudinal versus cross-sectional design, plasma/blood versus serum, and distinct outcome scales. The multi-metal odds-ratio range is not assigned to individual elements.
Antimony–ALT	Xie et al.: 5,548 adults; urinary antimony and prevalent metabolic-associated fatty liver disease, adjusted OR 1.18 (95% CI 1.08–1.29) per natural-log unit [29].	Index-based hepatic phenotype and urinary biomarker, not direct serum-antimony–ALT replication.
GGT non-findings	NHANES III: 10,098 adults; GGT increased across blood-lead and urinary-cadmium deciles [10]. Peng et al.: 281 adults; serum copper–GGT correlation r = 0.198 (p = 0.001), remaining positive after adjustment [28].	Lead and cadmium were not nominally associated with GGT in our serum analysis. Whole-blood lead and urinary cadmium are different biomarkers; the serum non-findings are not a contradiction or direct replication test of NHANES. None of the nominal GGT associations met the primary FDR criterion.
Iron/copper–bilirubin	Peng and Wei: 264 healthy adults; standardized coefficients +0.276 for iron, −0.304 for copper, and +0.312 for zinc [11].	Matching iron/copper directions; zinc evidence less clear in the present study. Standardized coefficients are not percentage differences.
Cobalt–ferritin	HUNT 2: 448 women; low ferritin accompanied higher blood cobalt [25]. A 234-adolescent study found corresponding serum and blood relationships [12].	Iron status may influence circulating cobalt. Population and direction of modeling differ; no proof of cobalt-induced iron depletion.
Iron–ferritin	Paired subgroup correlations were +0.65 in 20 normal-weight women and −0.46 in 22 women with obesity [24].	Small unadjusted correlations; demonstrates context dependence rather than an established interaction or SPES inflammatory mechanism.
Selenium–proteins	Selenium fractionation in 21 healthy people identified different protein-associated fractions [4]; a supplementation trial separated total-selenium response from functional biomarkers [26].	Binding, chemical form, and nutritional context differ. Additional albumin adjustment substantially attenuated the selenium–total-protein coefficient (Table S13), but did not make albumin and total protein independent outcomes. No selenium fractions or mediation effects were measured.
Selenium–total calcium	In 402 healthy women, total-calcium differences involved protein-bound calcium without equivalent ionized-calcium differences [27].	Not a selenium study. Our albumin-adjusted total-calcium estimate was attenuated (Table S13); neither study establishes a selenium association with ionized calcium or altered calcium regulation.
Antimony–potassium	Human phlebotomy research found potassium differences after preceding fist clenching [30]. No closely comparable community serum-antimony–potassium study was identified in the targeted search.	The antimony estimates depend on treatment of low concentrations. Neither experimental collection effects nor the existing six-element association assessment identifies the cause in this cohort. Independent sensitive measurement and specimen-quality documentation are needed.

https://doi.org/10.3390/ijms27209313

Association or question	Human evidence	Comparison and limitation
Iron–phosphate	Iron/FGF23 relationships differed between healthy controls and inherited hypophosphatemia [31]. Intravenous iron effects differed markedly by formulation [32].	Neither rare-disease physiology nor formulation-dependent treatment effects identify the mechanism of between-person serum-iron variation.

The table distinguishes close directional comparisons from indirect physiological or treatment evidence. It is not a systematic review, meta-analysis, or claim of independent replication within SPES. Each cited source appears in the main reference list; exposure matrix, population, and effect measure must be considered before comparing magnitude.

https://doi.org/10.3390/ijms27209313

Table S8a. Focused antimony quantification-status contrasts (Sensitivity Analysis 2).

Outcome	n	Quantified vs below-LOQ % (95% CI)	p	q
Alanine aminotransferase	4,126	+5.71 (+2.71 to +8.80)	<0.001	0.012
Potassium	4,126	+1.56 (+0.93 to +2.19)	<0.001	<0.001
Creatinine	4,126	−0.01 (−0.91 to +0.90)	0.981	0.994

The binary contrast compares quantified with below-LOQ observations among eligible available assays; it is distinct from the continuous per-log-IQR contrast. Lithium comparisons were not fitted because the below-LOQ group contained fewer than 50 observations, below the specified minimum group-size criterion.

Table S8b. Lithium–creatinine with additional total protein intake (Sensitivity Analysis 16).

Additional covariate	n	Adjusted % difference (95% CI)	p	q
Total protein intake (g/day)	4,131	+0.54 (+0.22 to +0.87)	0.001	0.020

The model adds total protein intake and does not adjust comprehensively for food sources or dietary patterns. Sensitivity Analysis 16 included no additional nutrient covariate for ALT or potassium.

Table S8c. Potassium associations with selected erythrocyte-enriched elements (Sensitivity Analysis 21).

Element	n	Potassium % difference (95% CI)	p	q
Cadmium	4,131	+0.11 (−0.32 to +0.54)	0.611	0.779
Copper	4,131	−0.03 (−0.23 to +0.17)	0.778	0.891
Iron (Fe-56)	4,131	+0.07 (−0.09 to +0.23)	0.399	0.608
Manganese	4,131	−0.01 (−0.11 to +0.09)	0.825	0.904
Lead	4,131	−0.22 (−0.47 to +0.03)	0.084	0.382
Zinc	4,131	−0.15 (−0.37 to +0.07)	0.170	0.504

The six-element assessment uses the primary potassium estimates and their original primary-family q values, without a separate six-test multiplicity adjustment or additional laboratory measurements. No positive association met the FDR criterion. The absence of a generalized positive association pattern does not exclude specimen-level preanalytical effects.

Table S9. Biochemical outcome definitions, units, and completion status.

Outcome	Analysis identifier	Source field	Unit	Observed n	Missing-outcome handling
Creatinine	creatinine	CREATININEMIA	mg/dL	4137	Observed
Alanine aminotransferase (ALT)	ALT	GPT (ALT)	U/L	4137	Observed
Gamma-glutamyl transferase (GGT)	GGT	Gamma G.T.	U/L	4137	Observed
Potassium	potassium	POTASSIO	mmol/L	4137	Observed
Urea	azotemia	AZOTEMIA	mg/dL	4137	Observed
Uric acid	uric_acid	URICEMIA	mg/dL	4137	Observed
Aspartate aminotransferase (AST)	AST	GOT (AST)	U/L	4137	Observed
Alkaline phosphatase	alkaline_phosphatase	FOSFATASI ALCALINA	U/L	4137	Observed
Total bilirubin	total_bilirubin	Bilirubina Totale	mg/dL	4137	Observed
Direct bilirubin	direct_bilirubin	Diretta	mg/dL	4137	Observed
Pseudocholinesterase	pseudocholinesterase	PSEUDOCOLINESTERASI	U/L	4137	Observed
Sodium	sodium	SODIEMIA	mmol/L	4137	Observed
Chloride	chloride	CLOREMIA	mmol/L	4137	Observed
Calcium	calcium	CALCIO (siero)	mg/dL	4137	Observed
Phosphate	phosphate	FOSFORO (siero)	mg/dL	4137	Observed
Albumin	albumin	Albumina	g/dL	4136	PMM for 1 missing value
Total protein	total_protein	ProteineTotali	g/dL	4136	PMM for 1 missing value
Beta-2 globulin fraction	beta2_globulin	Beta2 (g/dl)	g/dL	4136	PMM for 1 missing value
Gamma globulin fraction	gamma_globulin	Gamma (g/dl)	g/dL	4136	PMM for 1 missing value
Ferritin	ferritin	FERRITINA	ng/mL	4137	Observed

Outcomes were natural-log transformed after confirmation of finite positive values. Urea corresponds to the original azotemia identifier. Globulin measurements are electrophoretic-fraction concentrations rather than percentages; beta-2 globulin is distinct from beta-2 microglobulin. The source ALT/AST units of mU/mL are numerically equivalent to U/L. Sensitivity Analysis 12 restricts the four protein-related outcomes to their originally observed values. Selected observed outcome distributions are reported in main Table 1.

Table S10. Covariate dictionary for the primary Model B specification.

Covariate	Unit/categories	Model representation	Coding / interpretation
Age	Years	Restricted cubic spline	Knots 21, 30, 40, 48
Sex	Female; male	Female reference	Recorded sex; included in Model A/B
BMI	kg/m^2	Restricted cubic spline	Knots 20.37, 24.62, 31.14; weight/height/BMI data-validity checks in SM1
Smoking	Never; former; current; unknown	Never reference	Raw status 3 = never, 2 = former, 1 = current; positive cigarettes/day resolves missing status to current
Alcohol	g/day	ln(1 + value)	Questionnaire/dietary source
Physical activity	MET-h/week	ln(1 + value)	Questionnaire-derived activity
Total energy	kcal/day	ln(1 + value)	Dietary source; other nutrient terms limited to SA16
Deprivation	Recorded numerical score	Untransformed score	deprivation_score; omission sensitivity SA15
Potential occupational exposure	No; yes; unknown	No reference	Questionnaire-derived indicator, not measured workplace dose
Municipal cluster	21 geographic groups	Fixed-effect factor	Broad spatial adjustment; reference group 1
Municipality	Municipality of residence	CR2 covariance clustering	Residual dependence; not an additional dose or sampling weight

Model A includes age, sex, municipal-cluster fixed effects, and the scaled element. Model B additionally includes the remaining covariates. Unknown responses are retained as explicit categories. Age and BMI spline knots are fixed across outcome–element pairs. Alcohol, activity, and energy use log(1 + value), retaining zero values. The full-cohort sensitivity analysis uses Model A because questionnaire covariates were not imputed for the 51 participants without questionnaire data.

Table S11. Detailed sensitivity estimates for the three primary associations meeting the FDR criterion.

Association	Sensitivity module / analysis	n	Adjusted % difference (95% CI)	q
Lithium–creatinine	Primary	4,131	+0.54 (+0.22 to +0.87)	0.028
Lithium–creatinine	Alternative completion (SA4)	4,128	+0.57 (+0.24 to +0.90)	0.019
Lithium–creatinine	Outcome trimming (SA6)	4,100	+0.47 (+0.16 to +0.79)	0.048
Lithium–creatinine	Municipality random intercept (SA13)	4,131	+0.53 (+0.22 to +0.84)	0.036
Lithium–creatinine	Quantified only (SA3)	4,104	+0.70 (+0.32 to +1.08)	0.009
Lithium–creatinine	Huber point estimate (SA7)	4,131	+0.43	—
Antimony–ALT	Primary	4,131	+4.39 (+1.92 to +6.92)	0.020
Antimony–ALT	Alternative completion (SA4)	4,126	+2.31 (+0.87 to +3.77)	0.034
Antimony–ALT	Outcome-inclusive completion (SA19)	4,126	+2.23 (+0.82 to +3.67)	0.026
Antimony–ALT	Outcome trimming (SA6)	4,054	+4.07 (+1.86 to +6.33)	0.011
Antimony–ALT	Municipality random intercept (SA13)	4,131	+2.82 (+0.65 to +5.02)	0.199
Antimony–ALT	Quantified only (SA3)	2,622	−3.35 (−9.49 to +3.19)	0.564
Antimony–ALT	Huber point estimate (SA7)	4,131	+3.29	—
Antimony–potassium	Primary	4,131	+1.22 (+0.69 to +1.75)	<0.001
Antimony–potassium	Alternative completion (SA4)	4,126	+0.64 (+0.32 to +0.95)	0.003
Antimony–potassium	Outcome-inclusive completion (SA19)	4,126	+0.63 (+0.32 to +0.95)	0.003
Antimony–potassium	Outcome trimming (SA6)	4,107	+1.32 (+0.79 to +1.86)	<0.001
Antimony–potassium	Municipality random intercept (SA13)	4,131	+0.83 (+0.39 to +1.27)	0.019
Antimony–potassium	Quantified only (SA3)	2,622	−0.54 (−1.77 to +0.72)	0.639
Antimony–potassium	Huber point estimate (SA7)	4,131	+0.97	—
Lithium–creatinine	Protein-intake adjustment (SA16)	4,131	+0.54 (+0.22 to +0.87)	0.020
Antimony–ALT	Quantified vs below-LOQ status (SA2)	4,126	+5.71 (+2.71 to +8.80)	0.012
Antimony–potassium	Quantified vs below-LOQ status (SA2)	4,126	+1.56 (+0.93 to +2.19)	<0.001

Sensitivity Analysis 4/Sensitivity Analysis 19 used eligible samples held fixed across 60 completions, excluding three lithium or five antimony high-concentration plausibility records relative to primary Model B. Continuous models retain the fixed log-IQR scale; Sensitivity Analysis 2 uses a binary quantification-status contrast. Sensitivity Analysis 7 reports point estimates without inferential statistics. Reported q values retain their original testing-family definitions and were not recalculated across the selected results in this table. None of these supplementary continuous estimates has been relabeled as a per-doubling value.

Table S12. Sensitivity estimates for all 23 secondary/exploratory associations meeting their original family criteria.

Outcome / element	Original secondary estimate, % (95% CI)	Alternative completion, % (95% CI); n	Quantified only, % (95% CI); n
Uric acid / Lithium	+1.37 (+0.82 to +1.92)	+1.45 (+0.89 to +2.01); n=4,128	+2.02 (+1.36 to +2.70); n=4,104
Uric acid / Arsenic	+1.24 (+0.74 to +1.74)	+1.15 (+0.67 to +1.62); n=4,130	+2.68 (+1.68 to +3.69); n=3,835
Uric acid / Selenium	+0.91 (+0.31 to +1.52)	+0.89 (+0.28 to +1.51); n=4,131	+1.07 (+0.48 to +1.67); n=4,130
Uric acid / Strontium	+2.75 (+1.75 to +3.75)	+2.76 (+1.77 to +3.75); n=4,131	+3.13 (+2.28 to +3.98); n=4,130
Alkaline phosphatase / Copper	+1.57 (+0.63 to +2.52)	+1.64 (+0.74 to +2.55); n=4,128	+1.92 (+0.94 to +2.90); n=4,127
Alkaline phosphatase / Vanadium	−1.14 (−1.80 to −0.48)	−1.04 (−1.64 to −0.43); n=4,119	−1.08 (−2.43 to +0.30); n=3,650
Total bilirubin / Copper	−2.53 (−3.96 to −1.07)	−2.59 (−4.05 to −1.12); n=4,128	−2.64 (−4.32 to −0.92); n=4,127
Total bilirubin / Iron	+8.00 (+3.41 to +12.80)	+7.36 (+2.52 to +12.43); n=4,128	+13.11 (+10.34 to +15.95); n=4,120
Direct bilirubin / Copper	−2.40 (−3.73 to −1.05)	−2.47 (−3.82 to −1.09); n=4,128	−2.51 (−4.09 to −0.91); n=4,127
Direct bilirubin / Iron	+7.38 (+3.19 to +11.75)	+6.74 (+2.35 to +11.31); n=4,128	+11.99 (+9.35 to +14.69); n=4,120
Sodium / Thallium	−0.16 (−0.25 to −0.06)	−0.14 (−0.22 to −0.06); n=4,130	+0.10 (−0.14 to +0.35); n=3,067
Chloride / Beryllium	+0.19 (+0.08 to +0.30)	+0.08 (+0.03 to +0.13); n=4,131	+0.07 (−0.18 to +0.33); n=1,263
Chloride / Antimony	+0.62 (+0.41 to +0.83)	+0.32 (+0.20 to +0.44); n=4,126	−0.52 (−0.99 to −0.04); n=2,622
Chloride / Selenium	−0.12 (−0.20 to −0.03)	−0.11 (−0.19 to −0.03); n=4,131	−0.12 (−0.21 to −0.02); n=4,130
Chloride / Vanadium	−0.11 (−0.18 to −0.04)	−0.10 (−0.17 to −0.03); n=4,119	−0.14 (−0.28 to +0.00); n=3,650
Total calcium / Selenium	+0.32 (+0.20 to +0.43)	+0.31 (+0.19 to +0.43); n=4,131	+0.34 (+0.20 to +0.47); n=4,130
Phosphate / Cobalt	−0.43 (−0.64 to −0.21)	−0.47 (−0.70 to −0.24); n=4,128	−0.91 (−1.42 to −0.39); n=4,095
Phosphate / Iron	−1.25 (−1.63 to −0.87)	−1.15 (−1.58 to −0.72); n=4,128	−1.64 (−2.21 to −1.07); n=4,120
Phosphate / Zinc	−0.78 (−1.19 to −0.37)	−0.72 (−1.12 to −0.31); n=3,951	−0.78 (−1.25 to −0.32); n=3,950
Albumin / Selenium	+0.67 (+0.42 to +0.91)	+0.66 (+0.41 to +0.91); n=4,131	+0.73 (+0.48 to +0.99); n=4,130
Total protein / Selenium	+0.71 (+0.46 to +0.96)	+0.71 (+0.46 to +0.96); n=4,131	+0.80 (+0.58 to +1.02); n=4,130
Ferritin / Cobalt	−4.69 (−6.79 to −2.55)	−5.25 (−7.45 to −2.99); n=4,128	−9.85 (−13.01 to −6.58); n=4,095
Ferritin / Iron	+13.71 (+5.29 to +22.80)	+12.88 (+4.39 to +22.05); n=4,128	+19.45 (+11.71 to +27.72); n=4,120

All continuous estimates use the original fixed element-specific log-IQR, not the per-doubling display in main Table 4. Main-model q values are reported in main Table 4; the alternative-completion and quantified-only analyses retain their original sensitivity-family corrections, not a new correction across the displayed pairs. Confidence intervals are pointwise 95% intervals, not simultaneous multiplicity-adjusted intervals. In particular, the negative quantified-only antimony–chloride interval excludes zero but its original q value exceeds 0.05 (see SR3).

Table S13. Additional post hoc exploratory albumin-adjusted selenium sensitivity analyses.

Outcome	Original Model B, % (95% CI)	Model B + log(albumin), % (95% CI)	Log-coefficient reduction	Nominal p, adjusted model
Total calcium	+0.61 (+0.38 to +0.83)	+0.29 (+0.08 to +0.50)	53.1%	0.008
Total protein	+1.37 (+0.88 to +1.86)	+0.65 (+0.32 to +0.99)	52.1%	0.00014

All estimates are percentage differences in the named outcome per doubling of selenium. Each model used n = 4,131, 174 municipalities, and the same 30 completed datasets; the 21 geographical fixed effects and original Model B covariates were retained. Albumin was added as one natural-log continuous covariate; the outcome remained total calcium or total protein. Coefficients and CR2 variances were pooled using Rubin's rules and Barnard–Rubin degrees of freedom. Reduction = 100 × [1 − β(adjusted)/β(original)] on the common log scale; it is not mediation, variance explained, or a formal paired test. New p values are nominal and two-sided; intervals are pointwise 95% intervals. Original screening estimates and their FDR families were not replaced. No ionized or formula-corrected calcium was analyzed. See SM5.1 and SR5.

Supplementary Figures

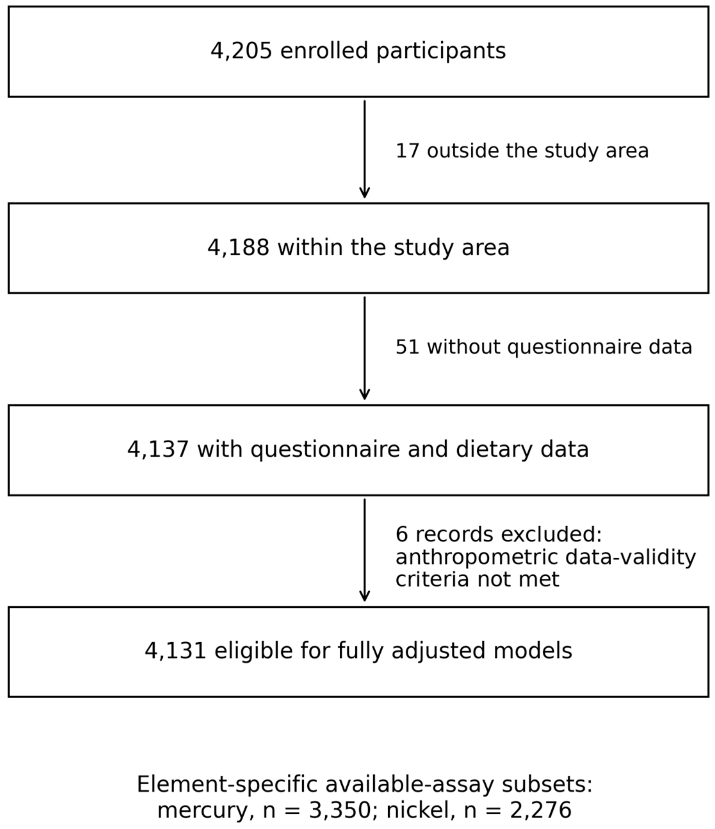

Figure S1. Inclusion of participants in the present analysis. Six records were excluded from fully adjusted models because weight, height, or calculated BMI failed the prespecified data-validity checks; these were analytical exclusions, not failures to meet SPES enrollment criteria. The 51 participants without questionnaire data were included only in the minimally adjusted full-cohort sensitivity. Mercury and nickel primary analyses used available-assay subsets.

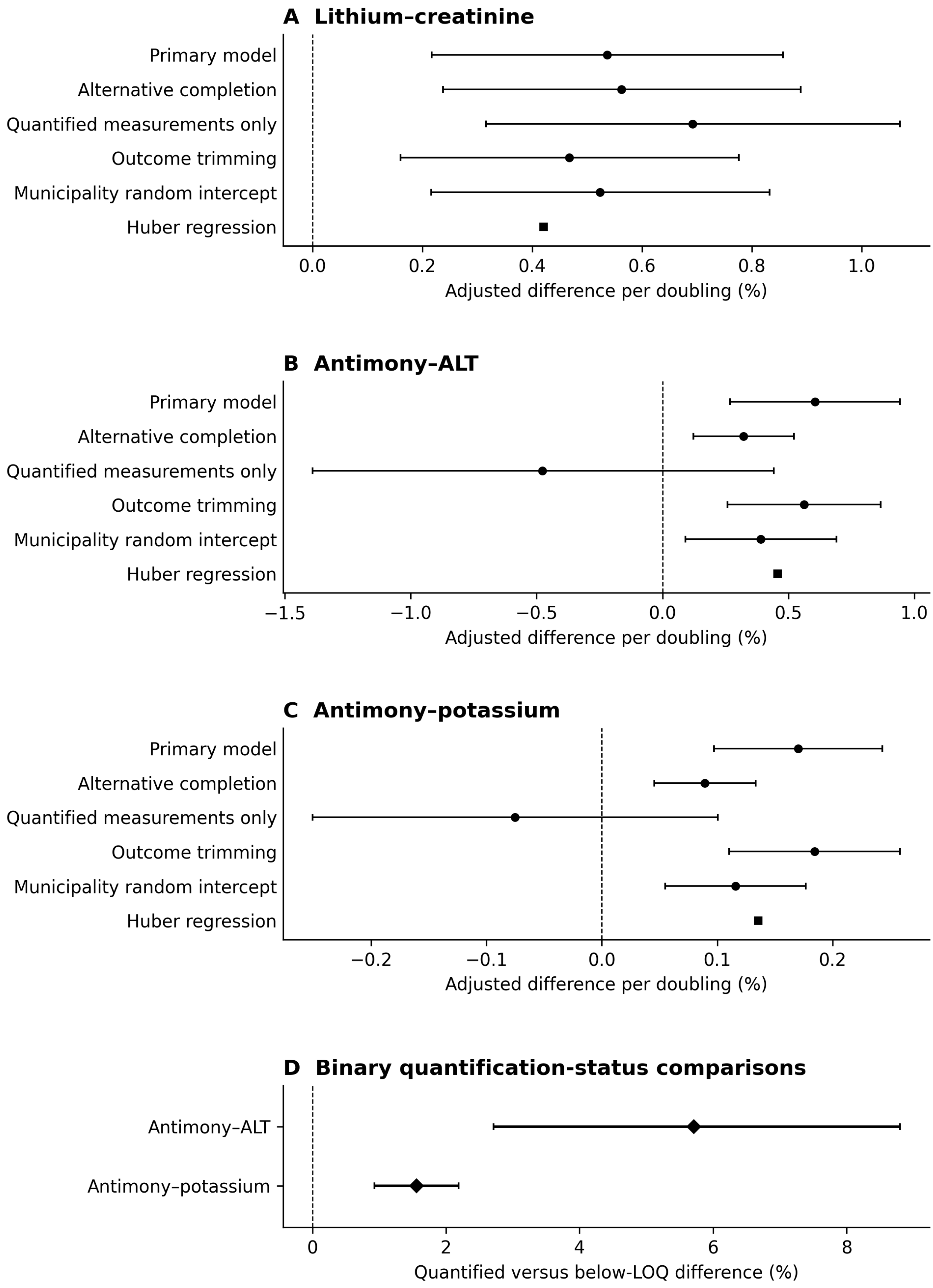

Figure S2. Selected sensitivity analyses. Panels A–C show continuous estimates per doubling of the relevant element; Panel D shows the separate binary contrast of quantified versus below-LOQ antimony. Points and bars indicate estimates and 95% confidence intervals; Huber squares have no inferential interval. Axes differ by panel. Eligibility and correction sets differ across specifications. Table S11 preserves the original fixed-log-IQR estimates and exact model denominators.

Disclaimer/Publisher's Note: The statements, opinions and data contained in all publications are solely those of the individual author(s) and contributor(s) and not of MDPI and/or the editor(s). MDPI and/or the editor(s) disclaim responsibility for any injury to people or property resulting from any ideas, methods, instructions or products referred to in the content.
